# Preparation of chiral 3-oxocycloalkanecarbonitrile and its derivatives by crystallization-induced diastereomer transformation of ketals with chiral 1,2-diphenylethane-1,2-diol†

**DOI:** 10.1039/c8ra06611f

**Published:** 2018-09-21

**Authors:** Yohei Yamashita, Daisuke Maki, Shiho Sakurai, Takumi Fuse, Shoji Matsumoto, Motohiro Akazome

**Affiliations:** Department of Applied Chemistry and Biotechnology, Graduate School of Engineering, Chiba University 1-33 Yayoicho, Inageku Chiba 263-8522 Japan akazome@faculty.chiba-u.jp; Process Chemistry Labs. Pharmaceutical Technology, Astellas Pharma Inc. 160-2, Akahama Takahagi-shi Ibaraki 318-0001 Japan yohei.yamashita@astellas.com; Molecular Chirality Research Center, Chiba University 1-33 Yayoicho, Inageku Chiba 263-8522 Japan

## Abstract

Chiral 3-oxocycloalkanecarbonitriles were prepared by fractional crystallization and crystallization-induced diastereomer transformation (CIDT) of diastereomeric ketals with (1*R*,2*R*)-1,2-diphenylethane-1,2-diol. Investigation of the crystal structures by X-ray diffraction analysis revealed that the difference in hydrogen bonds caused the discrepancy of the solubilities between (*R*) and (*S*) diastereomers. Furthermore, CIDT to afford the (*R*)-diastereomer in good yield (95% yield) and with high diastereoselectivity (97% de) was accomplished, which is the first example of CIDT of neutral compounds *via* formation of the diastereomeric ketal with (1*R*,2*R*)-1,2-diphenylethane-1,2-diol.

## Abstract 2

Chiral 3-oxocyclopentanecarbonitrile was prepared in good yield and with high diastereoselectivity by crystallization-induced diastereomer transformation of the corresponding diastereomeric ketal.

## Graphical abstract

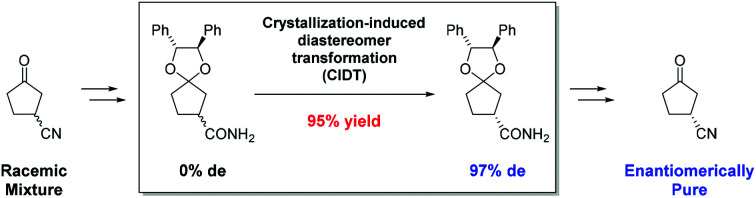

## Introduction

In recent years, the structure of active pharmaceutical ingredients and fine chemicals has become more complicated. To meet the demands for synthesizing organic molecules with a sophisticated design, building blocks containing chiral carbons as a component are widely used. Developing a new chiral building block will enable the synthesis of a new compound and provide benefits to both the chemical and pharmaceutical industries.

**Fig. 1:**
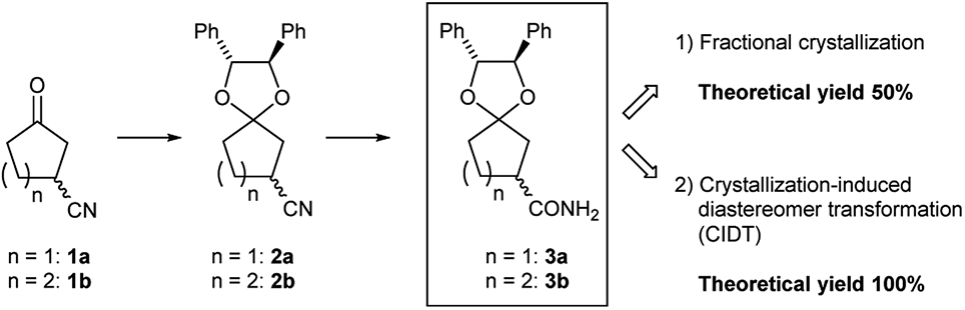
Chiral separation of racemic 3-oxocycloalkanecarbonitriles.

As new candidate building blocks, we focus on 3-oxocycloalkanecarbonitriles, 1a^1*a*,*b*^ and 1b^1*c*^ (Fig. 1). In fact, active pharmaceutical ingredients or intermediates containing 3-oxocycloalkanecarbonitriles or its derivatives have been reported,^2*a*,*b*^ which implies that 1 has potential as a building block. In spite of its structural simplicity, the preparation of enantiomerically pure 1 remains a challenging task.

Although 3-cyanoketones 1 are easily prepared from Michael addition of cyanide ions to α,β-unsaturated ketones,^3*a*,*b*^ there are a few articles reporting the preparation of chiral 3-cyanoketones by catalytic enantioselective conjugate addition of cyanide to enones. Specifically, 1b was obtained in 81% ee and 90% yield,^4^ and nucleophilic addition of formaldehyde dialkylhydrazones to conjugated enones has been reported.^5*a*,*b*^ However, no optical resolution of these neutral compounds has been reported, as these compounds are not applicable for diastereomeric salt separation, which is the most popular method to resolve racemic compounds. In fact, 3-oxocyclopentanecarboxylic acid as an acidic compound was resolved by diastereomeric salt formation with (−)-brucine, but four sequential crystallizations were required to obtain (*R*)-enantiomer in 98% ee.^6^

In order to introduce chiral moiety onto 1 and apply diastereomeric separation, we use chiral ketals as not only a protecting group but also chiral resolving auxiliary. Among several 1,2-diols, commercially available (1*R*,2*R*)- or (1*S*,2*S*)-1,2-diphenylethane-1,2-diol (dihydrobenzoin) shows great promise as a chiral auxiliary.^7^ In fact, several articles reported the separation of two isomers *via* formation of the diastereomeric ketals, and subsequent isolation either by column chromatography or crystallization.^8*a*–*c*^

**Fig. 2:**
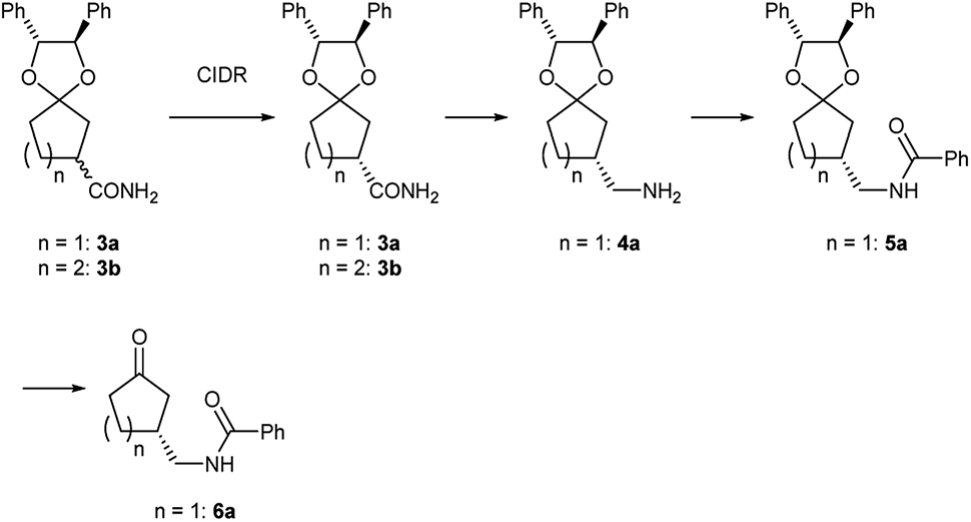
Functional group transformation of ketal 3.

Preliminarily, we synthesized diastereomeric mixtures of 2, but unfortunately, it was oily substance, which indicated that diastereomer separation by recrystallization was not applicable. Therefore, we transformed nitriles 2 into amides 3, which are generally expected to solidify due to the formation of hydrogen bonds. First, we examined the preparation of chiral 3-oxocycloalkanecarbonitrile and their derivatives *via* ketalization with (1*R*,2*R*)-1,2-diphenylethane-1,2-diol through fractional crystallization (Fig. 1). Even if separation of diastereomer is achieved through fractional crystallization, half of the diastereomeric mixture would remain as an undesired diastereomer. To our delight, 3 turned out to be racemized under basic conditions. Therefore, we performed crystallization-induced diastereomer transformation (CIDT)^9*a*–*f*^ on 3 and demonstrated the successful transformation of these compounds while keeping stereochemistry (Fig. 2).

## Results and discussion

### Synthesis of ketals

**Scheme 1:**
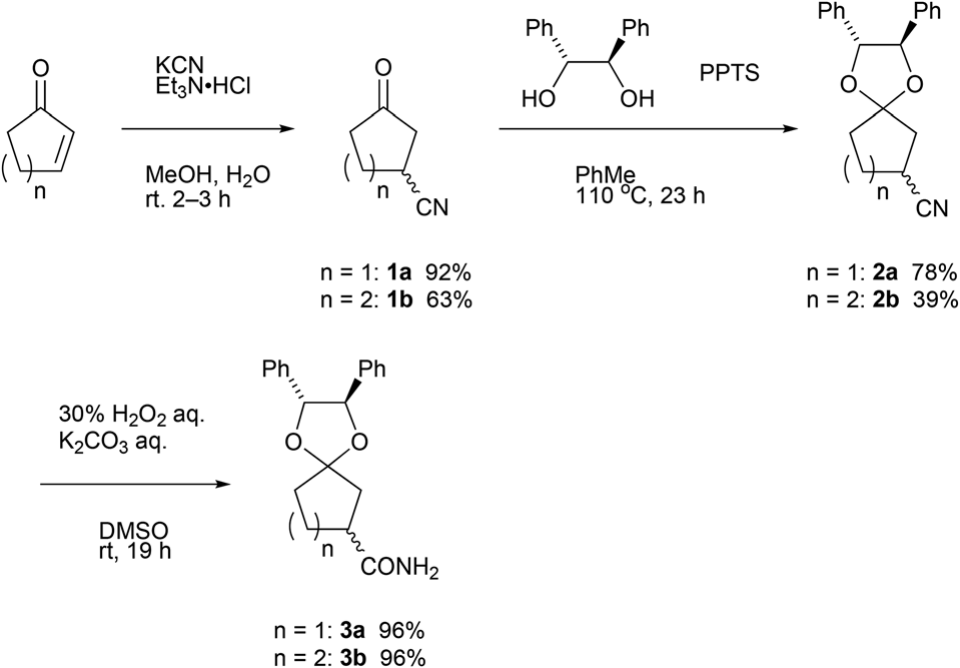
Synthesis of ketalized 3-oxocycloalkanecarbonitrile and 3-oxocycloalkanecarboxamide.

In accordance with the literature,^3*a*^ 3-cyanocyclopentanone 1a was prepared from Michael addition of cyanide ions to 2-cyclopentenone in 92%. In the case of 2-cyclohexenone, 3-cyanocyclohexanone 1b was obtained in lower yield (63%), but the value is comparable in the reported yield^3*b*^ (Scheme 1). Then, acid-catalyzed ketalization of 3-cyanocyclopentanone with 1,2-diphenylethane-1,2-diol was performed by pyridinium *p*-toluenesulfonate (PPTS).^10*a*,*b*^ Using an optimized reaction condition, namely, 1a (1.00 g, 9.20 mmol) with the diol (1.3 equiv.) and PPTS (0.1 equiv.) in toluene (30 mL) at 110 °C for 23 h, ketalization of 1a proceeded in 78% yield. Similarly, ketalization of 1b proceeded in 39% yield. Insufficient yield in ketalization of 1b is due to the contamination in 1b.^11^ Both diastereomeric mixtures of 2 were oily substance.

For the purpose of applying the diastereomer separation by fractional crystallization, we hydrated nitriles 2 with a combination of hydrogen peroxide and potassium carbonate^12^ to obtain crystalline amides 3.

### Fractional crystallization

To obtain a single diastereomer, we performed fractional crystallization on these amides 3 from the solution (toluene/CHCl_3_ = 2/3, Table 1). First, fractional crystallization of diastereomeric mixture 3a provided (*R*)-3a^13^ with 84% de (32% yield). An additional crystallization of the (*R*)-3a (84% de) achieved de of 99% (total yield 16%). In contract, first fractional crystallization (toluene/CHCl_3_ = 2/3) of diastereomeric mixture 3b was performed to obtain (*R*)-3b^13^ with only 30% de (46% yield). Second and third fractional crystallization (toluene/CHCl_3_ = 2/3) provided (*R*)-3b with 87% de and 99% de (total yield 14%). As shown in this result, both diastereomeric mixtures were separated by simple crystallization, and diastereomers of five-membered ketal 3a were more easily separated than those of six-membered 3b.

**Table 1:** Fractional crystallization of 3a and 3b^a^

Starting material		Crystallizations			
		None	1st	2nd	3rd
(*R*)-3a	% de^b^	3	84	>99	—
	Yield (%)	—	32	16	—
(*R*)-3b	% de^b^	10	30	87	>99
	Yield (%)	—	46	26	14

a In toluene/CHCl_3_ = 2/3.

b % de was determined by HPLC.

### Investigation of crystal structures by single-crystal X-ray diffraction analysis

To determine the stereochemistry of these diastereomers and to clarify why (*R*)-diastereomer crystallized preferably, we investigated the crystal structures of their diastereomers by single-crystal X-ray diffraction (SXRD) analysis. To obtain both diastereomers of 3, we first tried to separate the diastereomeric mixture of 3a by recycling preparative HPLC. However, 3a was inseparable due to the similar retention time of the diastereomers. In contrast, the corresponding nitriles 2a could be satisfactorily separated by recycling preparative HPLC. Here, we confirmed that both diastereomers of 2a were definitely oil. Then, (*R*)-2a and (*S*)-2a were transformed to crystalline amides (*R*)-3a and (*S*)-3a under the basic conditions mentioned above without epimerization.^12^ In sharp contrast with 2a, even ten cycles of recycling preparative HPLC could not separate 2b. After transformation to the corresponding amide 3b, diastereomeric mixtures of 3b were satisfactorily separated into (*R*)-3b and (*S*)-3b by recycling preparative HPLC.

**Fig. 3:**
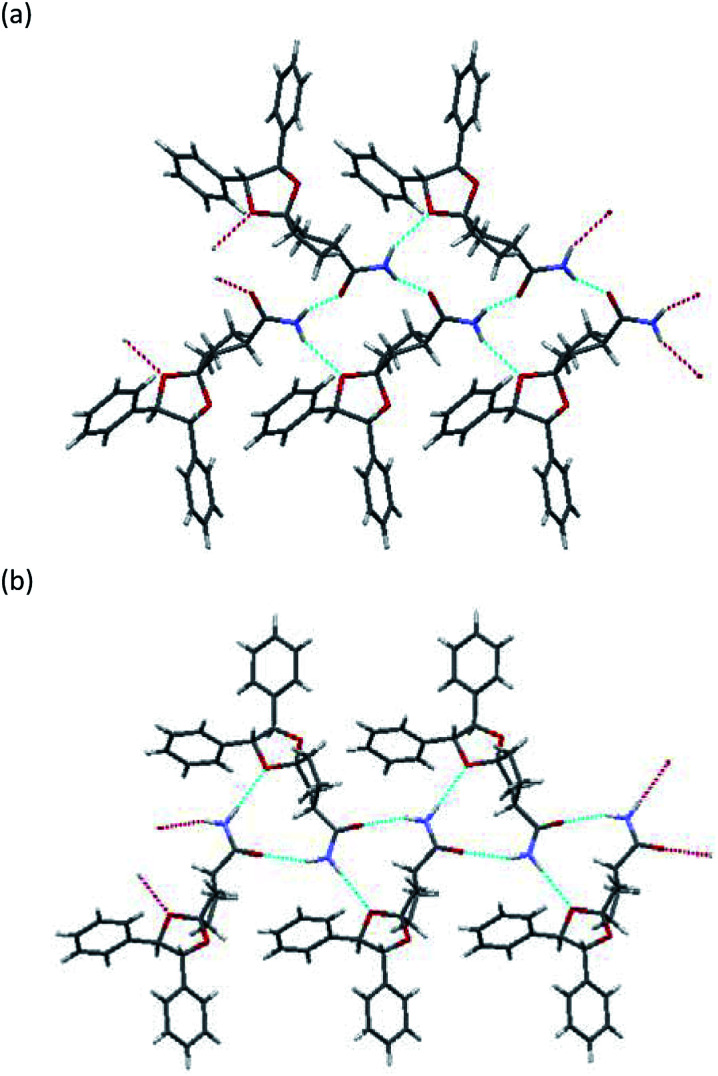
X-ray crystallographic structure: (a) (*R*)-3a, (b) (*S*)-3a.

**Fig. 4:**
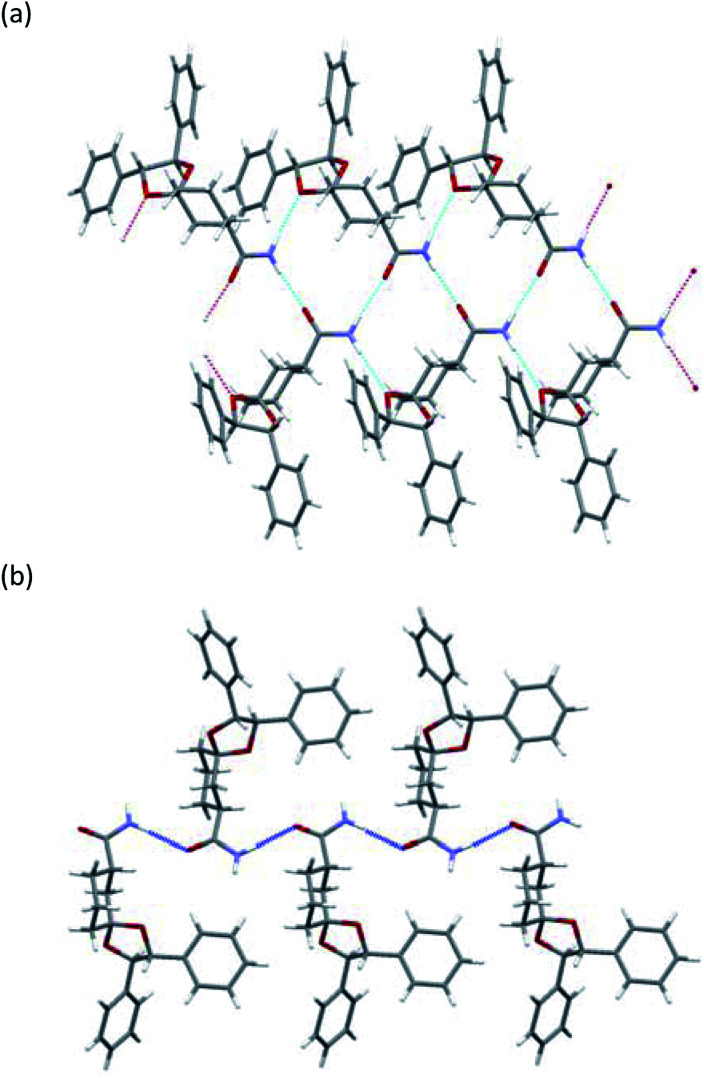
X-ray crystallographic structure: (a) (*R*)-3b, (b) (*S*)-3b.

All amides 3 were crystallized to obtain crystals suitable for SXRD analysis and we were able to determine the stereochemistry (Fig. 3 and 4). As shown in Fig. 3, both (*R*)-3a and (*S*)-3a had the same space group (*P*2_1_) and a similar molecular arrangement to construct hydrogen bonding networks. Their amide groups act as hydrogen donor and acceptor to construct the same number of hydrogen bonds, namely, two amide protons bound to two oxygen atoms of the amide and the ketal. While the cis proton against the carbonyl oxygen in (*R*)-3a constructed hydrogen bonds with the amide functional group, the trans one did in (*S*)-3a. The parameters of the hydrogen bonds in (*R*)-3a and (*S*)-3a are summarized in Table 2. The hydrogen bonding distances in (*R*)-3a crystals were shorter than those of (*S*)-3a. The crystals of (*R*)-3a (mp 139–140 °C, 1.26 g cm^−3^) had a higher melting point and larger calculated density than those of (*S*)-3a (mp 135–136 °C, 1.23 g cm^−3^). We performed solubility tests on each diastereomer in toluene at 25 °C and found that the values of (*R*)-3a and (*S*)-3a were 16.0 g L^−1^ and 21.5 g L^−1^, respectively, as anticipated. These results suggest that the difference of strength of the hydrogen bonds caused the discrepancy of the solubility and consequently enabled fractional crystallization providing (*R*)-3a.

**Table 2:** Hydrogen-bonding distances and angles of (*R*)-3a, (*S*)-3a, (*R*)-3b, and (*S*)-3b

	N⋯O_amide_ interaction		N⋯O_ketal_ interaction	
	N⋯O (H⋯O)/Å	N–H⋯O/°	N⋯O (H⋯O)/Å	N–H⋯O/°
(*R*)-3a	2.829 (1.983)	160.80	3.099 (2.295)	157.21
(*S*)-3a	3.195 (2.311)	151.76	3.239 (2.352)	178.40
(*R*)-3b	2.878 (2.001)	175.90	3.071 (2.194)	175.14
(*S*)-3b	2.917 (2.039)	176.77	—a	—a

a (*S*)-3b did not bind to an oxygen atom of the ketal.

As shown in Fig. 4, the crystals of (*R*)-3b and (*S*)-3b had space groups of *P*2_1_ and *P*2_1_2_1_2_1_, respectively. Similar molecular arrangements of constructing hydrogen bonding networks were observed in both diastereomers. While the cis proton against the carbonyl oxygen in (*R*)-3b constructed hydrogen bonds with amide functional groups, the cis one did not in (*S*)-3b. In other words, a cis proton of (*S*)-3b did not bind to an oxygen atom of the ketal. The crystals of (*R*)-3b (mp 164–165 °C) had a higher melting point than those of (*S*)-3b (mp 152–154 °C), but both had the same calculated density (1.24 g cm^−3^). Solubility tests on each diastereomer in toluene at 25 °C revealed that the values of (*R*)-3b and (*S*)-3b were 35.3 g L^−1^ and 49.0 g L^−1^, respectively. As discussed above, we conclude that the different hydrogen bonding networks caused the difference in both melting point and solubility and enabled fractional crystallization providing (*R*)-3b.

**Fig. 5:**
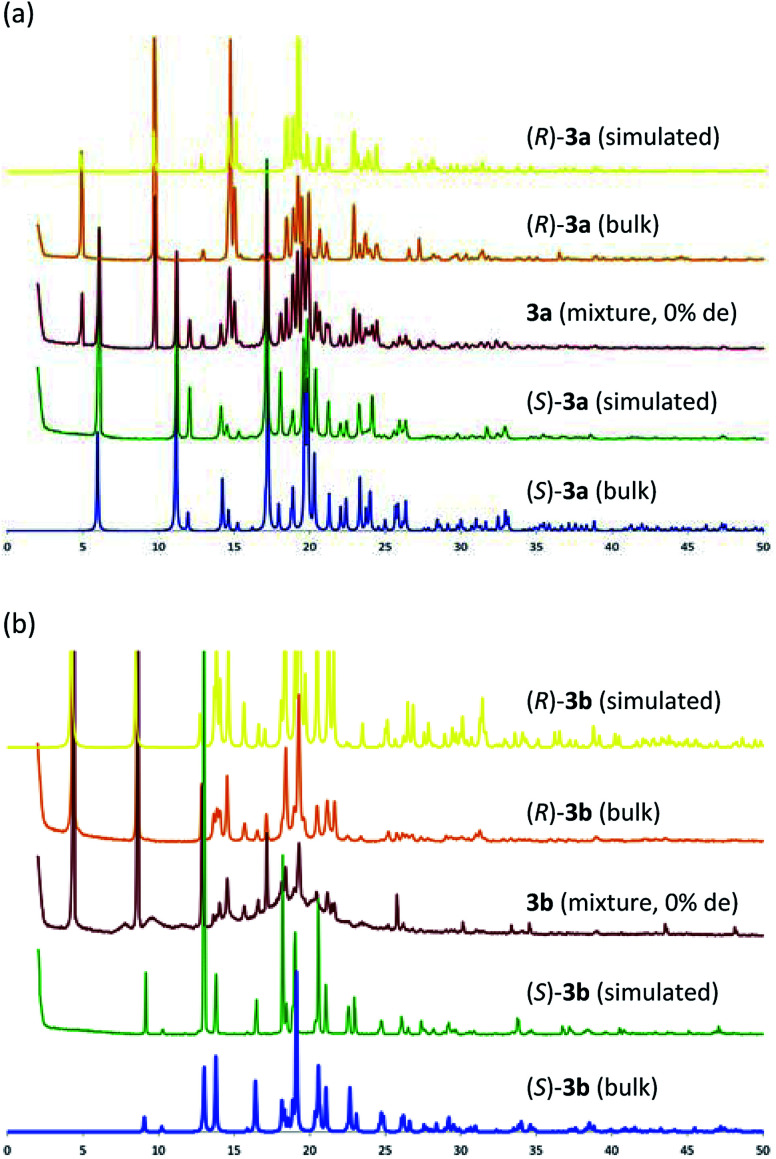
PXRD pattern: (a) 3a, (b) 3b.

Table 1 shows that the separation capacity of 3a is superior to that of 3b in the aspect of fractional crystallization. To elucidate the reason for this difference, both diastereomeric mixtures (3a and 3b) were crystallized simply from the solution (toluene/CHCl_3_ = 2/3) and the precipitated solid was analyzed using powder X-ray diffraction (PXRD) analysis. Fig. 5 shows the PXRD patterns of the crystallized diastereomeric mixture of 3 along with those of each single diastereomer as well as their simulated patterns calculated from SXRD. In the case of 3a, the PXRD pattern showed a superposition pattern of each diastereomer ((*R*)-3a and (*S*)-3a), which means the two diastereomers deposited separately. In contrast, the PXRD pattern of the solid precipitation of 3b shows a broad pattern with a partial component of (*R*)-3b. The pattern of (*S*)-3b was particularly hard to identify. These results suggest that the mixture of (*R*)-3b and (*S*)-3b might exist as an amorphous material and be what caused the broad PXRD pattern. If so, it would explain why the separation capacity of 3a is superior to that of 3b.

### Epimerization and crystallization-induced diastereomer transformation (CIDT)

Even if a racemic compound is optically resolved by the diastereomer method, half of the undesired diastereomer remains in the filtrate. Therefore, epimerization of the remaining diastereomer (*S*)-3 into (*R*)-3 is desirable from the viewpoint of yield. The screening of the epimerization conditions using (*S*)-3a and (*S*)-3b is summarized in Table 3. DBU in toluene and potassium *t*-butoxide in dioxane or THF were not effective (entries 1–3). (*S*)-3a (>99% de) was smoothly epimerized with potassium *t*-butoxide (2 equiv.) at 50 °C for 3.5 h in *t*-butanol, and the opposite diastereomer (*R*)-3a was slightly enriched with 7% de (entry 4). Under the same conditions, epimerization of (*S*)-3b (>99% de) proceeded more slowly than (*S*)-3a, where the % de of (*S*)-3b was 20% even after 7 h (entry 5). Other bases such as NaH and KOH were not effective (entries 6 and 7). The combination of potassium *t*-butoxide and *t*-butanol for CIDT has been reported,^14^ so we consider strong basic and protic conditions is appropriate for this epimerization. This positive result encouraged us to apply CIDT (Table 4). The treatment of 3a (0.25 mmol, 2% de) with potassium *t*-butoxide (0.5 equiv.) in *t*-butanol (0.20 mL) at room temperature precipitated (*R*)-3a with 80% de (87% yield, entry 1). An increased amount of *t*-butanol (0.40 mL) with an extended stirring time (96 h) improved % de to 97% (95% yield, entry 2). Meanwhile, a similar procedure to entry 2 using 3b precipitated (*R*)-3b with 14% de (85% yield, entry 3). Although the attempt to increase the reaction temperature to 80 °C in order to accelerate CIDT with addition of i-octane as a poor solvent provided better % de (44% de and 51% de, entries 4 and 5), these figures were not as high as those of 3a. As with the fractional crystallization of 3, the CIDT of 3a was superior to 3b. Anyway, we are convinced that the ketal moiety acted as not only a protecting group but also a chiral resolving auxiliary which is a useful tool for CIDT.

**Table 3:** Screening of epimerization conditions of 3^a^

	Entry	Base	Solvent	Temp. (°C)	Time (h)	% de^b^
1	(*S*)-3a	DBU	Toluene	110	24	98
2	(*S*)-3a	*t*-BuOK	Dioxane	100	24	98
3	(*S*)-3a	*t*-BuOK	THF	50	48	87
4	(*S*)-3a	*t*-BuOK	*t*-BuOH	50	3.5	−7
5	(*S*)-3b	*t*-BuOK	*t*-BuOH	50	7	20
6	(*S*)-3b	NaH	THF	50	24	96
7	(*S*)-3b	KOH	EtOH	50	8	88

a Conditions: (*S*)-3a or (*S*)-3b (>99% de, 0.050 mmol), base (2.0 equiv.), and solvent (1.5 mL) was used.

b % de was determined by HPLC.

**Table 4:** Crystallization-induced diastereomer transformation (CIDT) of 3^a^

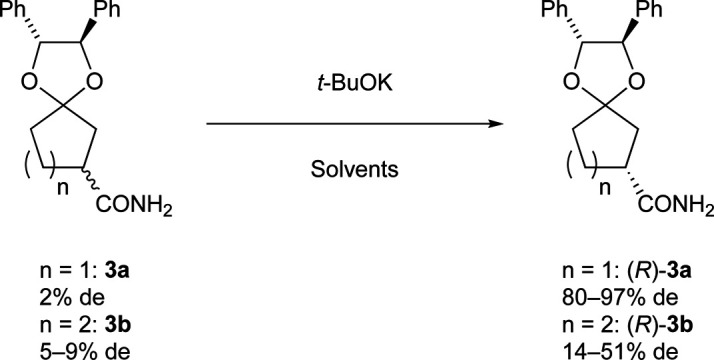							
	Entry (% de)	*t*-BuOK (equiv.)	Solvent (mL)	Temp. (^o^C)	Time (h)	(*R*)-3	
						% de^b^	Yield (%)
1	3a (2)	0.50	*t*-BuOH 0.20	rt	30	80	87
2	3a (2)	0.50	*t*-BuOH 0.40	rt	96	97	95
3	3b (5)	0.50	*t*-BuOH 0.40	rt	40	14	85
4	3b (9)	0.25	*t*-BuOH 0.05	80	96	44	75
			i-octane 0.25				
5	3b (9)	0.25	*t*-BuOH 0.10	80	72	51	54
			i-octane 0.70				

a 0.25 mmol scale.

b % de was determined by HPLC.

### Deprotection and derivatization of ketals

**Scheme 2:**
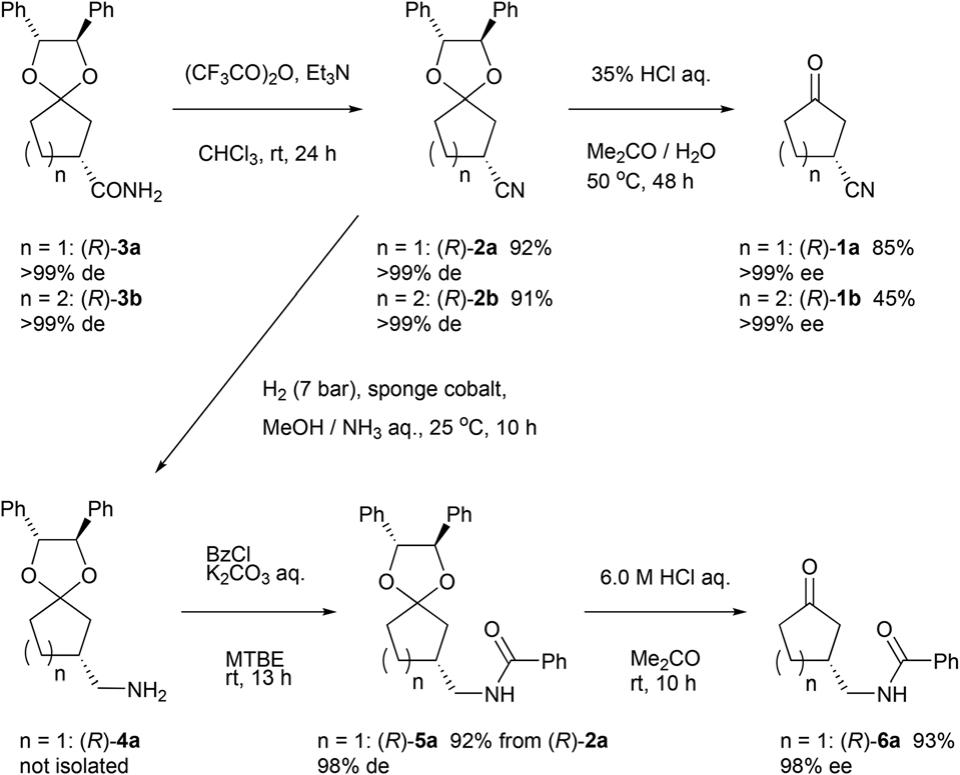
Synthesis of 3-oxocyclopentanecarbonitrile and its derivatives.

In order to show synthetic applications, derivatization of (*R*)-3a and (*R*)-3b was demonstrated (Scheme 2). (*R*)-3a and (*R*)-3b were dehydrated into nitrile (*R*)-2a and (*R*)-2b by treatment with trifluoroacetic anhydride^15^ and triethylamine in 92% and 91% yield, respectively. Subsequent deprotection of ketal groups by usual acidic condition provided 3-oxocycloalkanecarbonitriles (*R*)-1a and (*R*)-1b without epimerization.

Meanwhile, (*R*)-2a was reduced by hydrogenation using sponge cobalt^16^ to give primary amine (*R*)-4a which was immediately converted into benzoylated derivative (*R*)-5a. Deprotected ketone (*R*)-6a was finally obtained by usual acidic condition in 93% yield with 98% ee.

## Conclusions

We have synthesized chiral 3-oxocycloalkanecarbonitrile and 3-oxocycloalkanecarboxamide by fractional crystallization of ketal derivatives with (1*R*,2*R*)-1,2-diphenylethane-1,2-diol. Investigation of the crystal structures by X-ray diffraction analysis revealed that the difference in hydrogen bonds caused the discrepancy of the solubilities between the (*R*) and (*S*) diastereomers. Furthermore, CIDT to obtain (*R*)-3a in good yield (95% yield) and with high diastereoselectivity (97% de) was accomplished. Finally, successful derivatization of functional group and deprotection of ketals were performed without epimerization. To the best of our knowledge, we demonstrated the first example of CIDT of neutral compounds *via* formation of the diastereomeric ketal with (1*R*,2*R*)-1,2-diphenylethane-1,2-diol. These findings can be applied for synthesizing chiral and neutral building blocks containing carbonyl group.

## Experimental section

### General information

Starting materials, reagents, and solvents were obtained from commercial suppliers and used without further purification. Optical rotations were measured with a JASCO DIP-140 digital polarimeter at 20 °C using the sodium D line, and optical rotation data were reported as follows: [*α*]^20^_D_ (concentration *c* = g/100 mL, solvent). ^1^H and ^13^C NMR spectra were acquired with a Varian Gemini 2000 NMR spectrometer at 300 MHz and 75 MHz, respectively. Chemical shifts (*δ*) of ^1^H NMR were expressed in parts per million (ppm) relative to tetramethylsilane (*δ* = 0) as an internal standard. Multiplicities are indicated as br (broadened), s (singlet), d (doublet) and m (multiplet), and coupling constants (*J*) are reported in Hz unit. Chemical shifts (*δ*) of ^13^C NMR were expressed in ppm downfield or upfield from CDCl_3_ as an internal standard (*δ* = 77.0). Infrared spectra were acquired using in KBr disk with a JASCO FT/IR-460 plus spectrometer. Mass spectra were acquired with a Thermo Fisher Scientific Exactive spectrometer. Powder X-ray diffraction were acquired with a Bruker D8 ADVANCE. Single-crystal X-ray diffraction were acquired with Bruker APEX II and Bruker APEXII Ultra CCD diffractometers. Recycling preparative HPLC was performed with a JAI LC-908. Enantiomeric excess (ee) and diastereomeric excess (de) were determined by chiral HPLC analysis with a JASCO LC-2000Plus system and a SHIMAZU LC-2010 system.

### (2*R*,3*R*)-2,3-Diphenyl-1,4-dioxaspiro[4.4]nonane-7-carbonitrile (2a)

Potassium cyanide (3.86 g, 59.3 mmol) and triethylamine hydrochloride (8.24 g, 59.9 mmol) were dissolved in a mixed solution of methanol (12 mL) and water (10 mL). A solution of cyclopenten-2-one (4.17 g, 50.8 mmol) in methanol (8 mL) was added dropwise over 30 min at rt. The reaction mixture was stirred at rt for 2 h, and then acidified with 4 M HCl aqueous solution. After extraction with CHCl_3_ (20 mL × 5), the combined organic layer was dried over MgSO_4_, filtered and concentrated under vacuum to give 1a (5.10 g, 46.7 mmol, 92% yield). A solution of 1a (1.00 g, 9.20 mmol) in toluene (30 mL) was added pyridinium *p*-toluenesulfonate (230 mg, 0.920 mmol) and (1*R*,2*R*)-1,2-diphenylethane-1,2-diol (2.54 g, 11.9 mmol). The mixture was stirred at 110 °C in a Dean–Stark apparatus for 23 h. After cooling, the reaction mixture was washed with 5 wt% NaHCO_3_ aqueous solution (10 mL). The organic layer was dried over MgSO_4_, filtered and concentrated under vacuum. Toluene (30 mL) was added and the precipitated solid was separated by filtration. The filtrate was concentrated under vacuum and purified by silica gel column chromatography (*n*-heptane–AcOEt) to give 2a (2.17 g, 7.10 mmol, 78% yield from 1a) as a diastereomeric mixture. ^1^H NMR (CDCl_3_) *δ* 7.34–7.32 (m, 6H), 7.22–7.17 (m, 4H), 4.76–4.65 (m, 2H), 3.14–2.92 (m, 1H), 2.60–2.26 (m, 4H), 2.23–2.04 (m, 2H); ^13^C NMR (CDCl_3_) *δ* 136.1, 136.0, 135.8, 135.7, 128.49, 128.46, 128.4, 126.64, 126.60, 126.5, 126.4, 122.2, 121.9, 116.77, 116.72, 85.6, 85.5, 85.4, 41.7, 41.4, 36.5, 36.2, 28.3, 28.0, 25.54, 25.45; HRMS (ESI) *m*/*z* [M + NH_4_]^+^ calcd for C_20_H_23_N_2_O_2_ 323.1760, found 323.1751.

### (2*R*,3*R*,7*R*)-2,3-Diphenyl-1,4-dioxaspiro[4.4]nonane-7-carbonitrile ((*R*)-2a) and (2*R*,3*R*,7*S*)-2,3-diphenyl-1,4-dioxaspiro[4.4]nonane-7-carbonitrile ((*S*)-2a)

2a (301 mg, 0.986 mmol) was separated into (*R*)-2a (90.0 mg, 0.295 mmol, 30% yield) and (*S*)-2a (100 mg, 0.327 mmol, 33% yield) by recycling preparative HPLC (column: SiO_2_, eluent: *n*-hexane/AcOEt = 3/1). (*R*)-2a: mp 71–73 °C; [*α*]^20^_D_ + 19.1 (*c* = 1.00, CHCl_3_); ^1^H NMR (CDCl_3_) *δ* 7.34–7.32 (m, 6H), 7.22–7.17 (m, 4H), 4.72 (d, *J* = 8.5 Hz, 1H), 4.66 (d, *J* = 8.5 Hz, 1H), 3.02–2.91 (m, 1H), 2.55–2.41 (m, 2H), 2.37–2.25 (m, 2H), 2.23–2.04 (m, 2H); ^13^C NMR (CDCl_3_) *δ* 136.1, 135.8, 128.61, 128.55, 128.52, 128.49, 126.7, 126.4, 121.9, 116.8, 85.64, 85.58, 41.8, 36.6, 28.4, 25.6; FTIR (KBr, cm^−1^) 3035, 2989, 2950, 2909, 2238, 1718, 1605, 1493, 1456, 1434; HRMS (ESI) *m*/*z* [M + NH_4_]^+^ calcd for C_20_H_23_N_2_O_2_ 323.1760, found 323.1754. (*S*)-2a: colorless oil; [*α*]^20^_D_ + 68.4 (*c* = 1.00, CHCl_3_); ^1^H NMR (CDCl_3_) *δ* 7.35–7.31 (m, 6H), 7.22–7.19 (m, 4H), 4.75 (d, *J* = 8.5 Hz, 1H), 4.70 (d, *J* = 8.5 Hz, 1H), 3.15–3.04 (m, 1H), 2.60–2.53 (m, 1H), 2.42–2.05 (m, 5H); ^13^C NMR (CDCl_3_) *δ* 136.2, 135.9, 128.60, 128.57, 128.49 (large intensity), 126.7, 126.5, 122.3, 116.8, 85.6, 85.5, 41.5, 36.3, 28.1, 25.5; FTIR (KBr, cm^−1^) 3064, 3033, 2982, 2947, 2885, 2239, 1750, 1605, 1496, 1456; HRMS (ESI) *m*/*z* [M + NH_4_]^+^ calcd for C_20_H_23_N_2_O_2_ 323.1760, found 323.1754.

### (2*R*,3*R*)-2,3-Diphenyl-1,4-dioxaspiro[4.5]decane-7-carbonitrile (2b)

Potassium cyanide (3.56 g, 54.6 mmol) and triethylamine hydrochloride (8.25 g, 59.9 mmol) were dissolved in a mixed solution of methanol (14 mL) and water (10 mL). A solution of cyclohex-2-en-1-one (4.83 g, 50.2 mmol) in methanol (6.0 mL) was added over 30 min at rt. The reaction mixture was stirred at rt for 3 h, and then acidified with 4 M HCl aqueous solution. After extraction with CHCl_3_ (20 mL × 5), the combined organic layer was dried over MgSO_4_, filtered and concentrated under vacuum to give 1b (3.86 g, 31.3 mmol, 63% yield) as a diastereomeric mixture. A solution of 1b (990 mg, 8.04 mmol) in toluene (10 mL) was added pyridinium *p*-toluenesulfonate (200 mg, 0.796 mmol) and (1*R*,2*R*)-1,2-diphenylethane-1,2-diol (2.60 g, 12.1 mmol). The mixture was stirred at 110 °C in a Dean–Stark apparatus for 23 h. After cooling, the precipitated solid was separated by filtration. The filtrate was concentrated under vacuum and purified by silica gel column chromatography (*n*-heptane–AcOEt) to give 2b (1.01 g, 3.16 mmol, 39% yield from 1b) as a diastereomeric mixture. ^1^H NMR (CDCl_3_) *δ* 7.36–7.31 (m, 6H), 7.22–7.19 (m, 4H), 4.80–4.70 (m, 2H), 3.03–2.88 (m, 1H), 2.45–2.37 (m, 1H), 2.11–1.58 (m, 7H); ^13^C NMR (CDCl_3_) *δ* 136.0, 135.9, 135.8, 128.6, 128.54, 128.47, 128.4, 126.70, 126.65, 126.6, 126.5, 122.0, 121.9, 107.43, 107.41, 85.4, 85.34, 85.26, 39.7, 38.7, 35.9, 35.0, 28.64, 28.57, 26.5, 26.1, 22.4, 22.0; HRMS (ESI) *m*/*z* [M + Na]^+^ calcd for C_21_H_21_NO_2_Na 342.1470, found 342.1454.

### (2*R*,3*R*)-2,3-Diphenyl-1,4-dioxaspiro[4.4]nonane-7-carboxamide (3a)

A solution of 2a (307 mg, 1.01 mmol) in dimethylsulfoxide (8 mL) was added 30 wt% H_2_O_2_ aqueous solution (0.7 mL) and K_2_CO_3_ (554 mg, 4.01 mmol) in ice bath. The reaction mixture was stirred at rt for 19 h, followed by addition of water for quenching. After extraction with CHCl_3_ (15 mL × 3), the combined organic layer was dried over MgSO_4_, filtered and concentrated under vacuum. The residue was crystallized with a mixed solution of acetone and *n*-hexane to give 3a (314 mg, 0.971 mmol, 96% yield) as a diastereomeric mixture. ^1^H NMR (CDCl_3_) *δ* 7.34–7.31 (m, 6H), 7.24–7.19 (m, 4H), 5.85–5.49 (m, 2H), 4.78–4.67 (m, 2H), 3.04–2.83 (m, 1H), 2.47–1.97 (m, 6H); ^13^C NMR (CDCl_3_) *δ* 177.8, 177.3, 136.5, 136.3, 128.5, 128.4, 126.74, 126.71, 126.5, 118.34, 118.27, 85.6, 85.5, 42.9, 42.5, 41.0, 40.6, 37.1, 36.8, 27.6, 27.5; HRMS (ESI) *m*/*z* [M + H]^+^ calcd for C_20_H_22_NO_3_ 324.1600, found 324.1593.

### (2*R*,3*R*,7*R*)-2,3-Diphenyl-1,4-dioxaspiro[4.4]nonane-7-carboxamide ((*R*)-3a)

(*R*)-3a was prepared in 88% yield (121 mg, 0.374 mmol, >99% de) from (*R*)-2a (130 mg, 0.425 mmol, >99% de) according to the procedure similar to that mentioned in 3a. Mp 139–140 °C; [*α*]^20^_D_ + 23.2 (*c* = 0.99, CHCl_3_); ^1^H NMR (CDCl_3_) *δ* 7.35–7.31 (m, 6H), 7.24–7.19 (m, 4H), 5.70 (brs, 1H), 5.35 (brs, 1H), 4.73 (d, *J* = 8.5 Hz, 1H), 4.69 (d, *J* = 8.5 Hz, 1H), 2.95–2.84 (m, 1H), 2.47–1.96 (m, 6H); ^13^C NMR (CDCl_3_) *δ* 177.3, 136.5, 136.3, 128.5 (large intensity), 128.3, 126.7, 126.5, 118.3, 85.59, 85.56, 42.9, 41.0, 37.1, 27.6; FTIR (KBr, cm^−1^) 3447, 3206, 3028, 2896, 1699, 1655, 1496, 1452, 1435, 1335; HRMS (ESI) *m*/*z* [M + H]^+^ calcd for C_20_H_22_NO_3_ 324.1600, found 324.1593; HPLC condition, CHIRALCEL OJ-H 250 mm × 4.6 mm, 5 μm, *n*-hexane/2-propanol = 90/10, flow rate 1.0 mL min^−1^, at 25 °C, wavelength 254 nm, retention times (*R*)-3a 20.3 min, (*S*)-3a 28.4 min.

### (2*R*,3*R*,7*S*)-2,3-Diphenyl-1,4-dioxaspiro[4.4]nonane-7-carboxamide ((*S*)-3a)

(*S*)-3a was prepared in 90% yield (135 mg, 0.417 mmol, >99% de) from (*S*)-2a (141 mg, 0.461 mmol, 99% de) according to the procedure similar to that mentioned in 3a. Mp 135–136 °C; [*α*]^20^_D_ + 47.9 (*c* = 1.00, CHCl_3_); ^1^H NMR (CDCl_3_) *δ* 7.34–7.31 (m, 6H), 7.24–7.19 (m, 4H), 5.84 (brs, 1H), 5.40 (brs, 1H), 4.77 (d, *J* = 8.5 Hz, 1H), 4.72 (d, *J* = 8.5 Hz, 1H), 3.04–2.93 (m, 1H), 2.46–2.04 (m, 6H); ^13^C NMR (CDCl_3_) *δ* 177.7, 136.6, 136.3, 128.49 (large intensity), 128.45, 128.3, 126.7, 126.5, 118.4, 85.5 (large intensity), 42.6, 40.6, 36.8, 27.5; FTIR (KBr, cm^−1^) 3455, 3352, 3031, 2979, 1670, 1607, 1456, 1439, 1334, 1122; HRMS (ESI) *m*/*z* [M + H]^+^ calcd for C_20_H_22_NO_3_ 324.1600, found 324.1594.

### (2*R*,3*R*)-2,3-Diphenyl-1,4-dioxaspiro[4.5]decane-7-carboxamide (3b)

3a was prepared in 96% yield (321 mg, 0.951 mmol) from 3b (317 mg, 0.939 mmol) according to the procedure similar to that mentioned in 3a. ^1^H NMR (CDCl_3_) *δ* 7.34–7.30 (m, 6H), 7.24–7.18 (m, 4H), 5.91–5.88 (m, 1H), 5.66–5.60 (m, 1H), 4.81–4.71 (m, 2H), 2.72–2.57 (m, 1H), 2.31–2.24 (m, 1H), 2.15–1.68 (m, 6H), 1.57–1.45 (m, 1H); ^13^C NMR (CDCl_3_) *δ* 177.5, 177.4, 136.5, 136.4, 136.3, 128.44, 128.43, 128.39, 128.3, 126.8, 126.7, 126.60, 126.58, 109.34, 109.30, 85.34, 85.29, 85.25, 85.1, 42.6, 42.1, 39.5, 38.6, 36.3, 35.3, 28.6, 28.4, 22.9, 22.5; HRMS (ESI) *m*/*z* [M + H]^+^ calcd for C_21_H_24_NO_3_ 338.1756, found 338.1741.

### (2*R*,3*R*,7*R*)-2,3-Diphenyl-1,4-dioxaspiro[4.4]decane-7-carboxamide ((*R*)-3b) and (2*R*,3*R*,7*S*)-2,3-diphenyl-1,4-dioxaspiro[4.4]decane-7-carboxamide ((*S*)-3b)

3b (151 mg, 0.448 mmol) was separated into (*R*)-3b (80 mg, 0.237 mmol, 53% yield) and (*S*)-3b (52 mg, 0.154 mmol, 34% yield) by recycling preparative HPLC (column: SiO_2_, eluent: *n*-hexane/AcOEt = 3/1). (*R*)-3b: mp 164–165 °C; [α] +19.0 (*c* = 1.00, CHCl_3_); ^1^H NMR (CDCl_3_) *δ* 7.36–7.29 (m, 6H), 7.23–7.18 (m, 4H), 5.64 (brs, 1H), 5.56 (brs, 1H), 4.78 (d, *J* = 8.5 Hz, 1H), 4.73 (d, *J* = 8.5 Hz, 1H), 2.68–2.58 (m, 1H), 2.29–2.24 (m, 1H), 2.16–1.70 (m, 6H), 1.60–1.46 (m, 1H); ^13^C NMR (CDCl_3_) *δ* 177.4, 136.42, 136.40, 128.5 (large intensity), 128.4, 128.3, 126.7, 126.6, 109.3, 85.4, 85.3, 42.6, 38.6, 36.3, 28.4, 22.5; FTIR (KBr, cm^−1^) 3433, 3209, 3031, 2948, 2876, 1687, 1664, 1497, 1341, 1164; HRMS (ESI) *m*/*z* [M + H]^+^ calcd for C_21_H_24_NO_3_ 338.1756, found 338.1751; HPLC condition, CHIRALCEL IB 250 mm × 4.6 mm, 5 μm, *n*-hexane/2-propanol = 16/1, flow rate 1.0 mL min^−1^, at 25 °C, wavelength 254 nm, retention times (*R*)-3b 21.2 min, (*S*)-3b 26.9 min. (*S*)-3b: mp 152–154 °C; [*α*]^20^_D_ + 69.1 (*c* = 1.00, CHCl_3_); ^1^H NMR (CDCl_3_) *δ* 7.34–7.30 (m, 6H), 7.24–7.19 (m, 4H), 5.57 (brs, 1H), 5.42 (brs, 1H), 4.80 (d, *J* = 8.5 Hz, 1H), 4.73 (d, *J* = 8.5 Hz, 1H), 2.73–2.65 (m, 1H), 2.32–2.27 (m, 1H), 2.09–1.68 (m, 6H), 1.59–1.48 (m, 1H); ^13^C NMR (CDCl_3_) *δ* 177.2, 136.6, 136.4, 128.50, 128.45, 128.4, 128.3, 126.9, 126.6, 109.4, 85.3, 85.1, 42.1, 39.6, 35.4, 28.6, 22.9; FTIR (KBr, cm^−1^) 3463, 3159, 2945, 1656, 1497, 1456, 1354, 1277, 1163, 1098; HRMS (ESI) *m*/*z* [M + H]^+^ calcd for C_21_H_24_NO_3_ 338.1756, Found 338.1751.

### Fractional crystallization to give (2*R*,3*R*,7*R*)-2,3-diphenyl-1,4-dioxaspiro[4.4]nonane-7-carboxamide ((*R*)-3a)

A solution of 3a (50 mg, 0.155 mmol) in a mixed solution of CHCl_3_ (0.6 mL) and toluene (0.4 mL) was left under slow evaporation conditions at rt for 2 d. The precipitated solid was collected by filtration. The procedure described above was repeated to give (*R*)-3a (8.0 mg, 0025 mmol, >99% de, 16% yield).

### Fractional crystallization to give (2*R*,3*R*,7*R*)-2,3-diphenyl-1,4-dioxaspiro[4.4]decane-7-carboxamide ((*R*)-3b)

A solution of 3b (50 mg, 0.148 mmol) in a mixed solution of CHCl_3_ (0.60 mL) and toluene (0.40 mL) was left slow evaporation conditions at rt for 3 d. The precipitated solid was collected by filtration. The procedure described above was repeated twice to give (*R*)-3b (7.0 mg, 0021 mmol, >99% de, 14% yield).

### Synthesis of (*R*)-3a by crystallization-induced diastereomer transformation (CIDT)

A mixture of 3a (76.0 mg, 0.235 mmol) and *t*-butanol (0.40 mL) was added potassium *t*-butoxide (14 mg, 0.125 mmol) in a sealed vial. The mixture was stirred at rt for 96 h. The precipitated solid was collected by filtration to give (*R*)-3a (72.0 mg, 0.223 mmol, 97% de, 95% yield).

### Synthesis of (*R*)-3b by CIDT

A mixture of 3b (81 mg, 0.240 mmol), *t*-butanol (0.10 mL) and i-octane (0.70 mL) was added potassium *t*-butoxide (7 mg, 0.062 mmol) in a sealed vial. The mixture was stirred at 80 °C for 72 h. The precipitated solid was collected by filtration to give (*R*)-3b (44.0 mg, 0.130 mmol, 51% de, 54% yield).

### Synthesis of (*R*)-2a by dehydration

A solution of (*R*)-3a (77 mg, 0.238 mmol, >99% de) in CHCl_3_ (2 mL) was added triethylamine (0.07 mL, 0502 mmol) and trifluoroacetic anhydride (0.04 mL, 0.284 mmol) in ice bath. The reaction mixture was stirred at rt for 3 h. Triethylamine (0.07 mL, 0502 mmol) and trifluoroacetic anhydride (0.04 mL, 0.284 mmol) were added to the reaction mixture in ice bath, and the mixture was stirred at rt for 18 h. After extraction with CHCl_3_ (10 mL × 3), the combined organic layer was washed with water (10 mL × 3), dried over MgSO_4_, filtered and concentrated under vacuum to give (*R*)-2a (67 mg, 0.219 mmol, yield 92%, >99% de).

### Synthesis of (*R*)-2b by dehydration

(*R*)-2b was prepared in 91% yield (69 mg, 0.216 mmol, >99% de) from (*R*)-3b (76 mg, 0.225 mmol, >99% de) according to the procedure similar to that mentioned in (*R*)-2a by dehydration.

### (*R*)-3-Oxocyclopentanecarbonitrile ((*R*)-1a)

A solution of (*R*)-2a (162 mg, 0.530 mmol, >99% de) in acetone (5 mL) was added 1.5 M HCl aqueous solution (1 mL). The reaction mixture was stirred at 50 °C for 48 h. After extraction with AcOEt (10 mL × 3), the combined organic layer was dried over MgSO_4_, filtered and concentrated under vacuum. The crude was purified by silica gel column chromatography (*n*-heptane–AcOEt) to give (*R*)-1a (49 mg, 0.449 mmol, 85% yield, >99% ee). [*α*]^20^_D_ + 41.7 (*c* = 1.00, CHCl_3_); ^1^H NMR (CDCl_3_) *δ* 3.26–3.15 (m, 1H), 2.68–2.42 (m, 4H), 2.38–2.21 (m, 2H); ^13^C NMR (CDCl_3_) *δ* 212.7, 120.8, 41.4, 36.7, 27.4, 25.6; FTIR (KBr, cm^−1^) 3480, 2984, 2921, 2243, 1747, 1461, 1405, 1152, 1141, 908; HRMS (ESI) *m*/*z* [M + H]^+^ calcd for C_6_H_8_NO 110.0606, found 110.0600. Spectral and analytical data were in agreement with the previous article.^5*a*^ Enantiomeric excess of (*R*)-1a was determined by the treatment with 2,4-dinitrophenylhydrazine hydrochloride.^17*a*,*b*^ HPLC condition, CHIRALCEL OD-RH 150 mm × 4.6 mm, 5 μm, elution A, 0.1% HClO_4_ aqueous solution, elution B, MeCN, gradient 50% A to 5% A over 25 min, flow rate 1.0 mL min^−1^, at 40 °C, wavelength 254 nm, retention times (*R*)-1a 11.6 min, (*S*)-1a 12.3 min.

### (*R*)-3-Oxocyclohexanecarbonitrile ((*R*)-1b)

(*R*)-1b was prepared in 45% yield (19 mg, 0.154 mmol, >99% de) from (*R*)-2b (110 mg, 0.344 mmol, >99% de) according to the procedure similar to that mentioned in (*R*)-1a. [*α*]^20^_D_ − 33.3 (*c* = 1.00, CHCl_3_); ^1^H NMR (CDCl_3_) *δ* 3.08–2.99 (m, 1H), 2.72–2.55 (m, 2H), 2.43–2.39 (m, 2H), 2.22–1.98 (m, 3H), 1.89–1.81 (m, 1H); ^13^C NMR (CDCl_3_) *δ* 205.3, 120.1, 43.2, 40.7, 28.6, 28.1, 23.7; FTIR (KBr, cm^−1^) 2957, 2873, 2241, 1718, 1451, 1419, 1362, 1325, 1261, 1225; HRMS (ESI) *m*/*z* [M − H]^−^ calcd for C_7_H_8_NO 122.0606, found 122.0605. HPLC condition, CHIRALPAK AS-H 250 mm × 4.6 mm, 5 μm, *n*-hexane/2-propanol = 2/1, flow rate 1.0 mL min^−1^, at 30 °C, wavelength 300 nm, retention times (*R*)-1b 11.1 min, (*S*)-1b 9.1 min.

### *N*-(((2*R*,3*R*,7*R*)-2,3-Diphenyl-1,4-dioxaspiro[4.4]nonan-7-yl)methyl)benzamide ((*R*)-5a)

A mixture of (*R*)-2a (400 mg, 1.31 mmol, 95% de), sponge cobalt (Nikko Rica R-400, 0.80 g), 28 wt% ammonia aqueous solution (0.80 mL) and methanol (3.2 mL) was stirred at 25 °C for 10 h under hydrogen atmosphere (7 bar) in an autoclave. The catalyst was removed by filtration and the filtrate was concentrated under vacuum followed by azeotropic distillation with methyl *t*-butyl ether (8.0 mL × 3) under vacuum to give (*R*)-4a. The mixture of (*R*)-4a, methyl *t*-butyl ether (8.0 mL) and 20 wt% K_2_CO_3_ aqueous solution (8.0 mL) were added benzoyl chloride (183 μl, 1.58 mmol) in ice bath, and the reaction mixture was stirred at rt for 13 h. After phase separation, the aqueous layer was extracted with methyl *t*-butyl ether (4.0 mL). The combined organic layer was dried over MgSO_4_, filtered and concentrated under vacuum. The crude was purified by silica gel column chromatography (*n*-heptane–AcOEt) to give (*R*)-5a (496 mg, 1.20 mmol, 92% yield, 98% de). Mp 129–130 °C; [*α*]^20^_D_ − 7.1 (*c* = 1.00, CHCl_3_); ^1^H NMR (CDCl_3_) *δ* 7.70–7.68 (m, 2H), 7.42–7.30 (m, 7H), 7.26–7.17 (m, 6H), 6.61 (brs, 1H), 4.74 (d, *J* = 8.5 Hz, 1H), 4.70 (d, *J* = 8.5 Hz, 1H), 3.65–3.45 (m, 2H), 2.60–2.51 (m, 1H), 2.44–2.36 (m, 1H), 2.24–1.94 (m, 4H), 1.68–1.54 (m, 1H); ^13^C NMR (CDCl_3_) *δ* 167.7, 136.6, 136.3, 134.4, 131.2, 128.6, 128.50, 128.44, 128.35, 128.3, 127.0, 126.8, 126.5, 118.5, 85.67, 85.65, 44.4, 41.6, 37.5, 36.1, 27.0; FTIR (KBr, cm^−1^) 3300, 3033, 2961, 2865, 1628, 1605, 1580, 1549, 1492, 1466; HRMS (ESI) *m*/*z* [M + H]^+^ calcd for C_27_H_28_NO_3_ 414.2069, found 414.2062; HPLC condition, CHIRALPAK IC 250 mm × 4.6 mm, 5 μm, *n*-hexane/ethanol = 90/10, flow rate 0.80 mL min^−1^, at 25 °C, wavelength 225 nm, retention times (*R*)-5a 13.8 min, (*S*)-5a 15.9 min.

### (*R*)-*N*-((3-Oxocyclopentyl)methyl)benzamide ((*R*)-6a)

A solution of (*R*)-5a (380 mg, 0.919 mmol, 98% de) in acetone (5.7 mL) was added 6.0 M HCl aqueous solution (184 μL). The reaction mixture was stirred at rt for 10 h. After concentration under vacuum, CHCl_3_ (7.6 mL) and 5 wt% NaHCO_3_ aqueous solution (3.8 mL) were added to the residue. The organic layer was concentrated under vacuum, and purified by silica gel column chromatography (*n*-heptane–AcOEt) to give (*R*)-6a (186 mg, 0.856 mmol, 93% yield, 98% ee). Mp 80–81 °C; [*α*]^20^_D_ + 71.5 (*c* = 1.00, CHCl_3_); ^1^H NMR (CDCl_3_) *δ* 7.78–7.75 (m, 2H), 7.55–7.42 (m, 3H), 6.44 (brs, 1H), 3.59 (dd, *J* = 13.5, 6.3 Hz, 1H), 3.51 (dd, *J* = 13.5, 6.3 Hz, 1H), 2.63–2.14 (m, 5H), 2.03–1.94 (m, 1H), 1.79–1.61 (m, 1H); ^13^C NMR (CDCl_3_) *δ* 218.2, 167.8, 134.3, 131.6, 128.6, 126.8, 44.0, 42.8, 38.0, 37.2, 27.0; FTIR (KBr, cm^−1^) 3271, 3080, 2959, 2932, 2872, 1739, 1630, 1601, 1577, 1553; HRMS (ESI) *m*/*z* [M + H]^+^ calcd for C_13_H_16_NO_2_ 218.1181, found 218.1176; HPLC condition, CHIRALPAK IC 250 mm × 4.6 mm, 5 μm, *n*-hexane/ethanol = 80/20, flow rate 0.80 mL min^−1^, at 25 °C, wavelength 225 nm, retention times (*R*)-6a 14.0 min, (*S*)-6a 16.6 min.

## Conflicts of interest

There are no conflicts of interest to declare.

## Supplementary Material

RA-008-C8RA06611F-s001

RA-008-C8RA06611F-s002
