# Supplementary material for: Preparation of chiral 3-oxocycloalkanecarbonitrile and its derivatives by crystallization-induced diastereomer transformation of ketals with chiral 1,2-diphenylethane-1,2-diol
Source: RSC Adv. 2018 Sep 21;8(57):32601–9. doi: 10.1039/c8ra06611f (PMC9086373; doi:10.1039/c8ra06611f)

Electronic Supplementary Material (ESI) for RSC Advances.
This journal is © The Royal Society of Chemistry 2018

Supplementary data for the article entitled

Preparation of chiral 3-oxocycloalkanecarbonitrile and its derivatives by crystallization-induced diastereomer transformation of ketals with chiral 1,2-diphenylethane-1,2-diol

Yohei Yamashita,*[a,b] Daisuke Maki,[a] Shiho Sakurai,[a] Takumi Fuse,[a] Shoji Matsumoto[a] and Motohiro Akazome*[a,c]

[a] *Department of Applied Chemistry and Biotechnology, Graduate School of Engineering, Chiba University, 1-33 Yayoicho, Inageku, Chiba 263-8522, Japan*

[b] *Process Chemistry Labs. Pharmaceutical Technology, Astellas Pharma Inc., 160-2, Akahama, Takahagi-shi, Ibaraki 318-0001, Japan*

[c] *Molecular Chirality Research Center, Chiba University, 1-33 Yayoicho, Inageku, Chiba, 263-8522, Japan.*

E-mail address: akazome@faculty.chiba-u.jp, yohei.yamashita@astellas.com

Table of Contents

1. ^{1}H and ^{13}C NMR spectra of **2a**, (*R*)-**2a**, (*S*)-**2a**, **2b**, **3a**, (*R*)-**3a**, (*S*)-**3a**, **3b**, (*R*)-**3b**, (*S*)-**3b**, (*R*)-**1a**, (*R*)-**1b**, (*R*)-**5a** and (*R*)-**6a** ………S2

2. Crystallographic data of (*R*)-**3a**, (*S*)-**3a**, (*R*)-**3b** and (*S*)-**3b** ..……S16

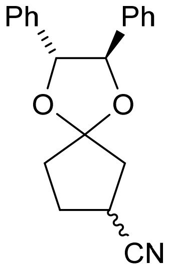
Ph
Ph
O
O
CN

2a

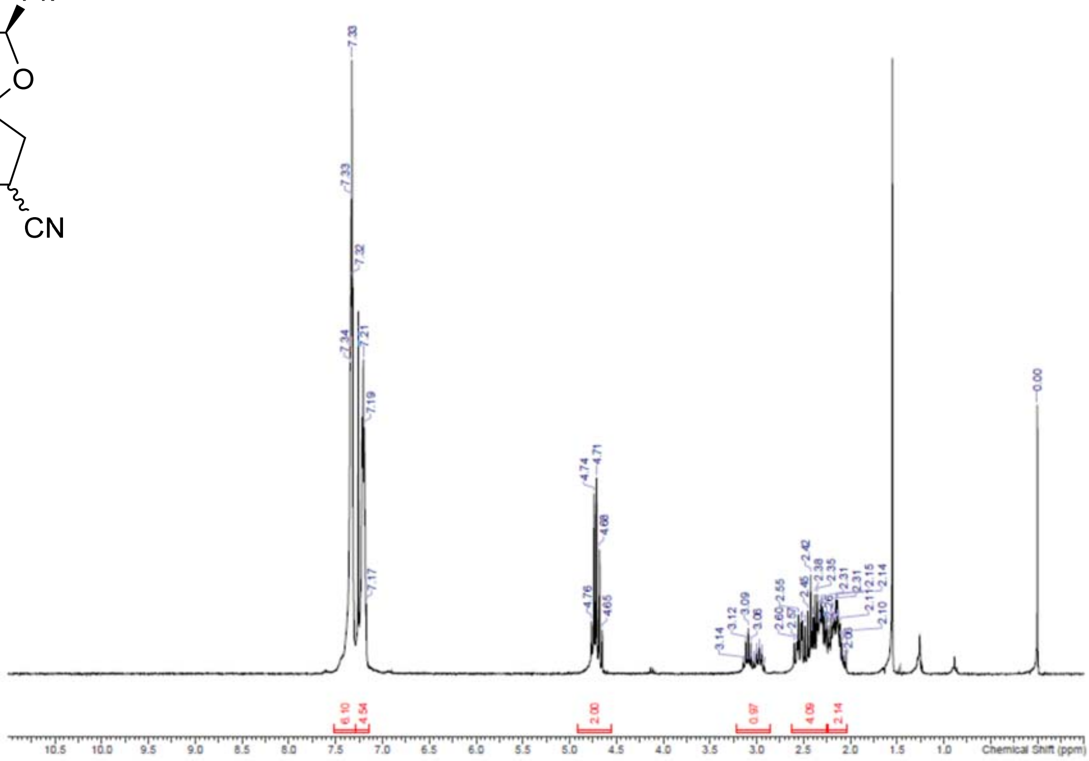
7.33
7.33
7.32
7.34
7.21
7.19
7.17
4.74
4.71
4.68
4.76
4.65
3.14
3.12
3.09
3.06
2.60
2.55
2.57
2.45
2.42
2.38
2.35
2.31
2.15
2.14
2.11
2.10
2.08
0.00
6.10
4.54
2.00
0.97
4.09
2.14
Chemical Shift (ppm)

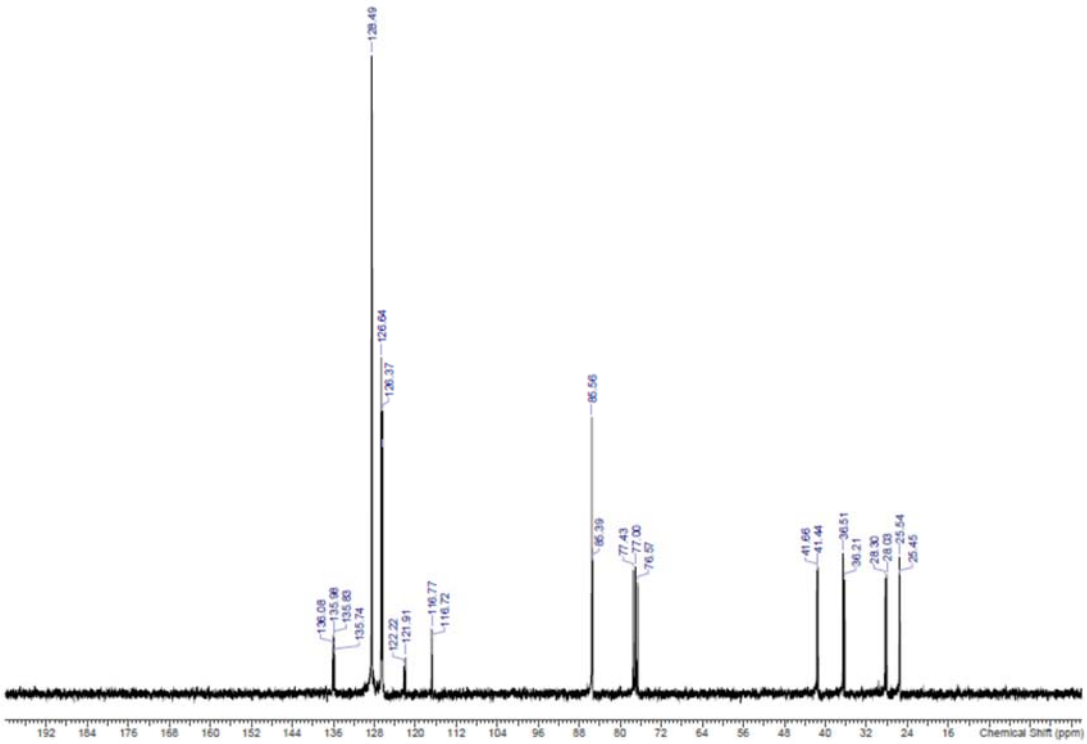
128.49
126.64
126.37
86.56
86.39
77.43
77.00
76.57
136.08
135.98
135.83
135.74
122.22
121.91
116.77
116.72
41.66
41.44
36.51
36.21
28.30
28.03
25.54
25.45
Chemical Shift (ppm)

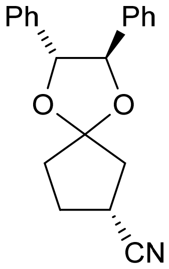

(*R*)-**2a**

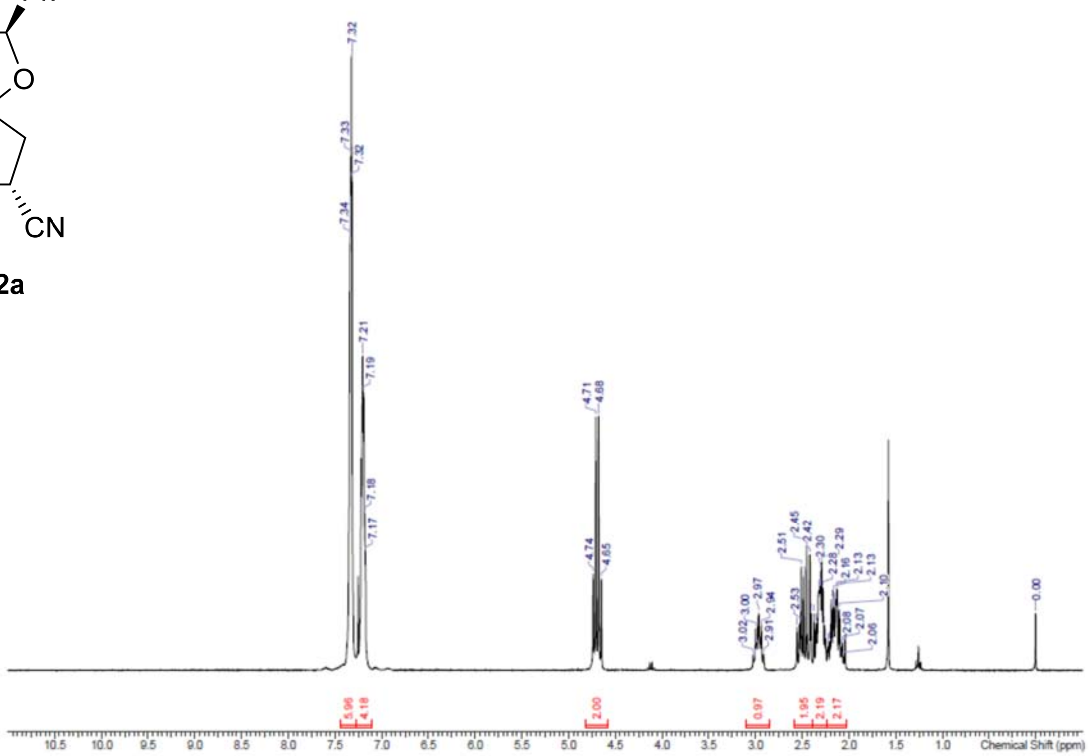

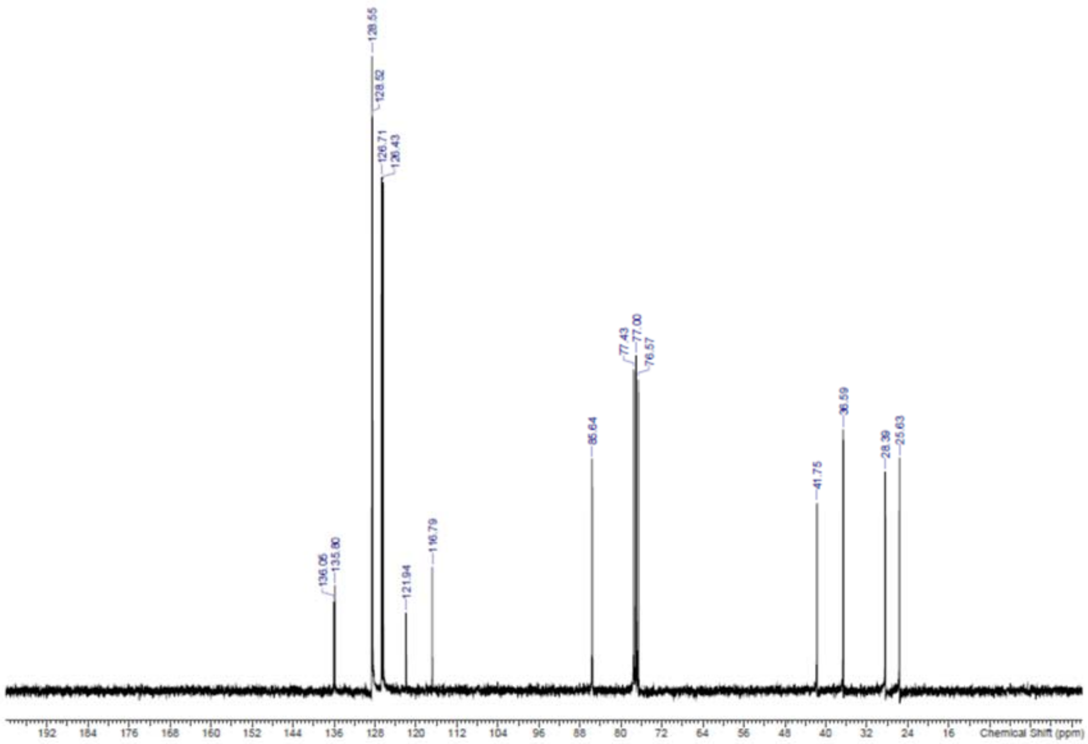

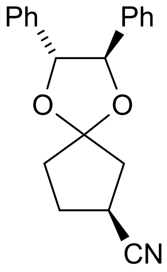
Ph
Ph
O
O
CN

(*S*)-**2a**

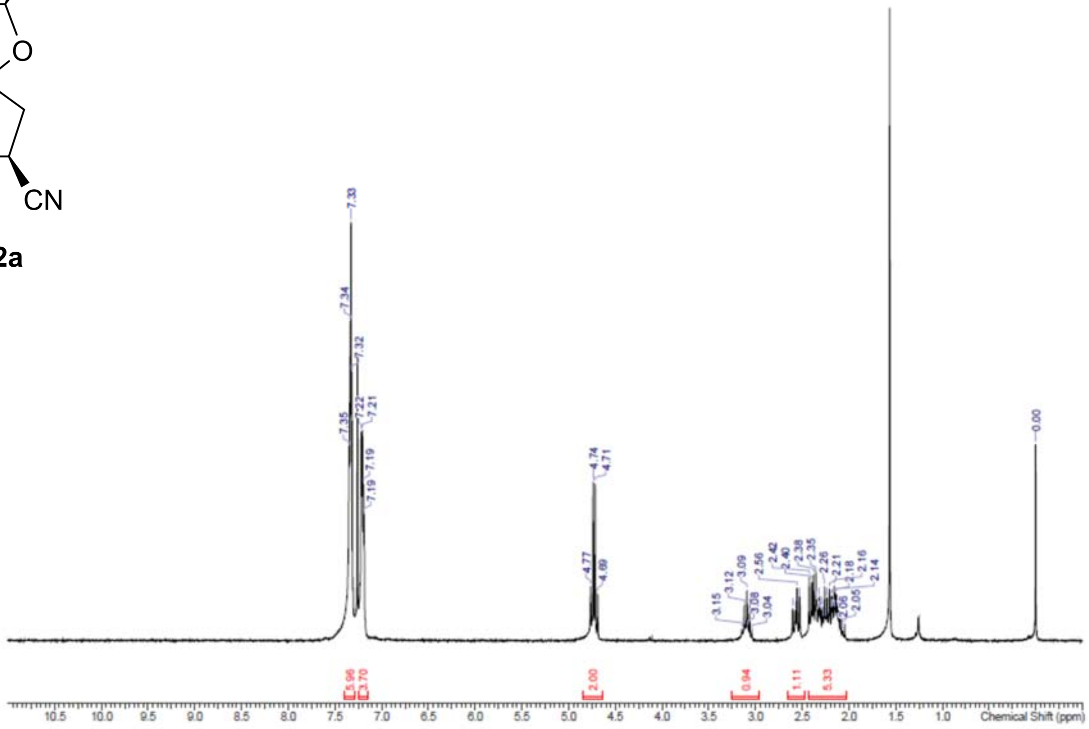
7.33
7.34
7.32
7.35
7.22
7.21
7.19
7.19
4.74
4.71
4.77
4.69
3.15
3.12
3.09
3.08
3.04
2.56
2.42
2.40
2.38
2.35
2.26
2.21
2.18
2.16
2.14
2.06
2.05
0.00
5.96
3.70
2.00
0.94
1.11
5.33
Chemical Shift (ppm)

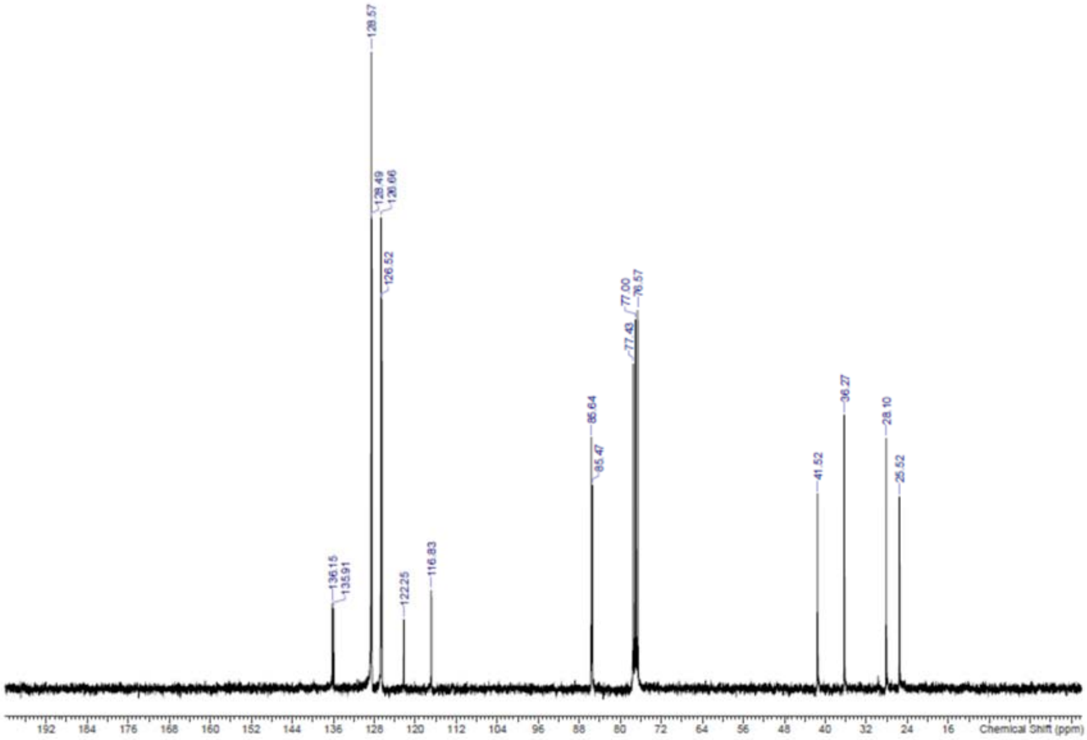
128.57
128.49
126.66
126.52
77.00
76.57
77.43
85.64
85.47
136.15
135.91
122.25
116.83
41.52
36.27
28.10
25.52
Chemical Shift (ppm)

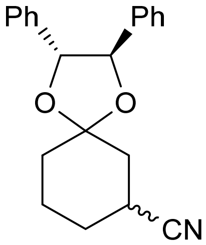

2b

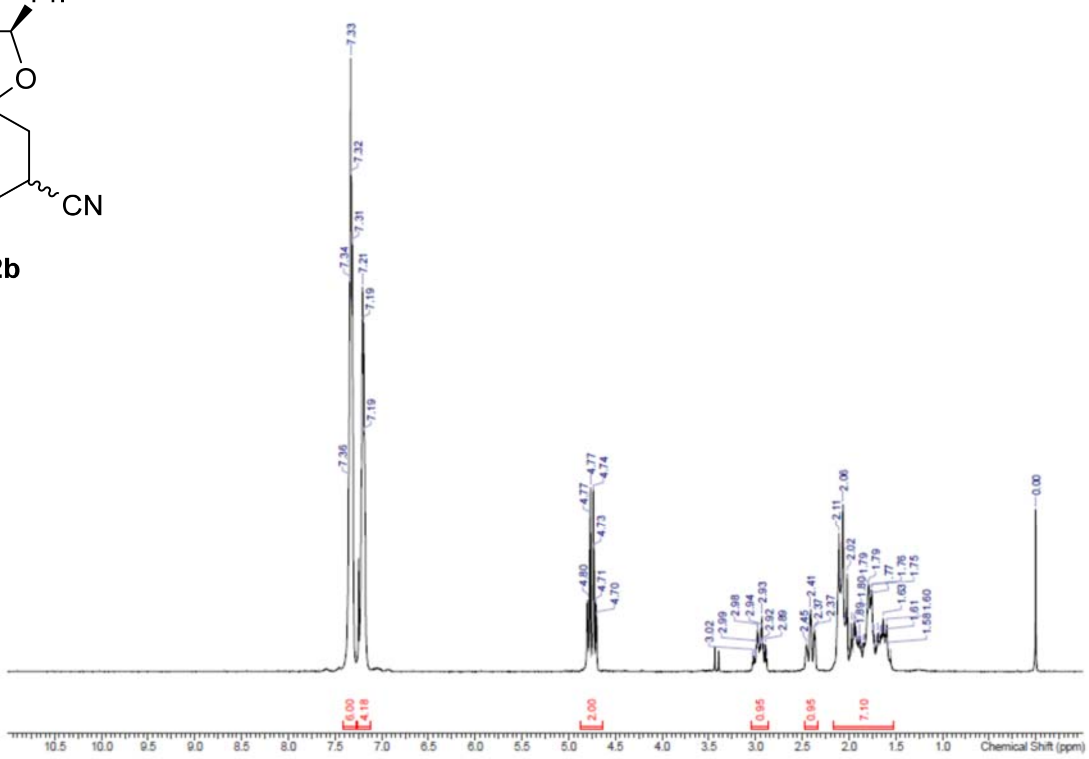

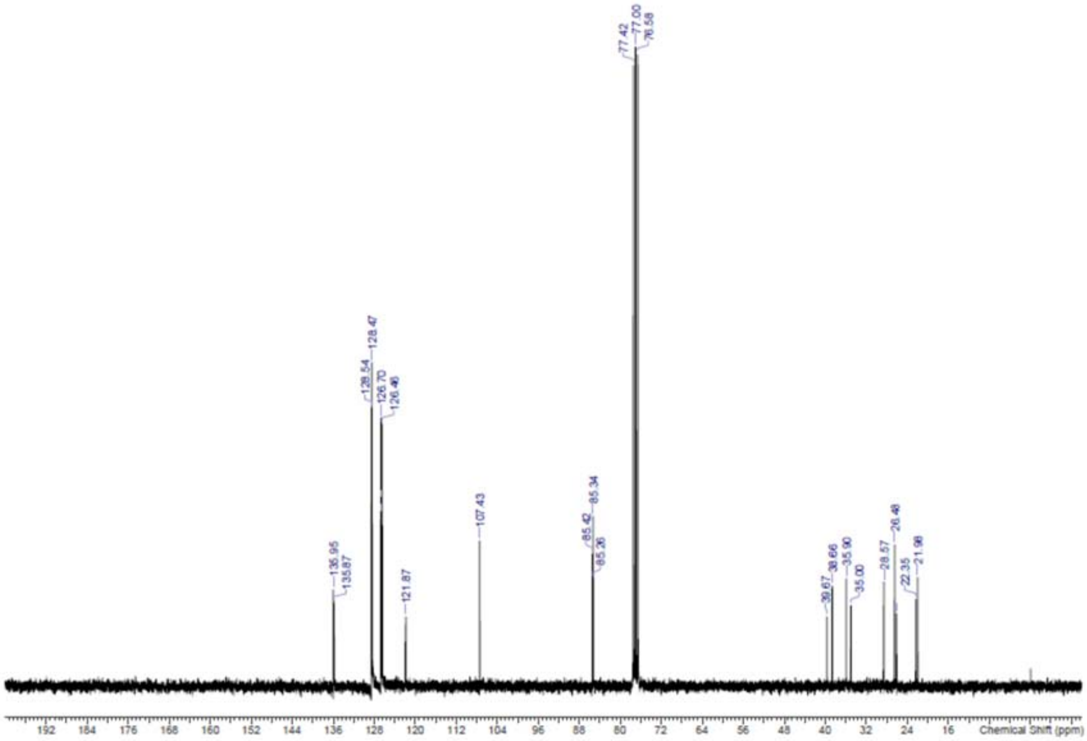

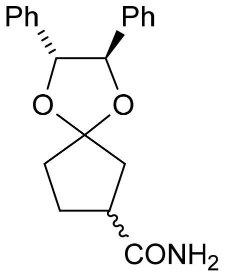

3a

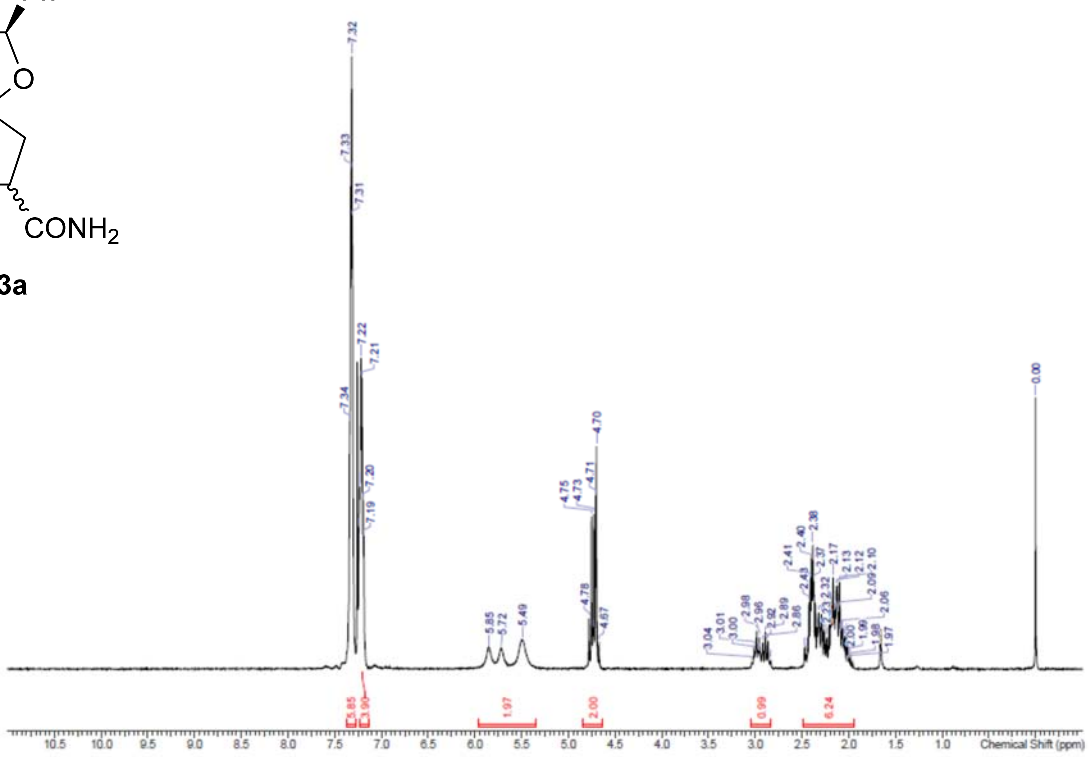

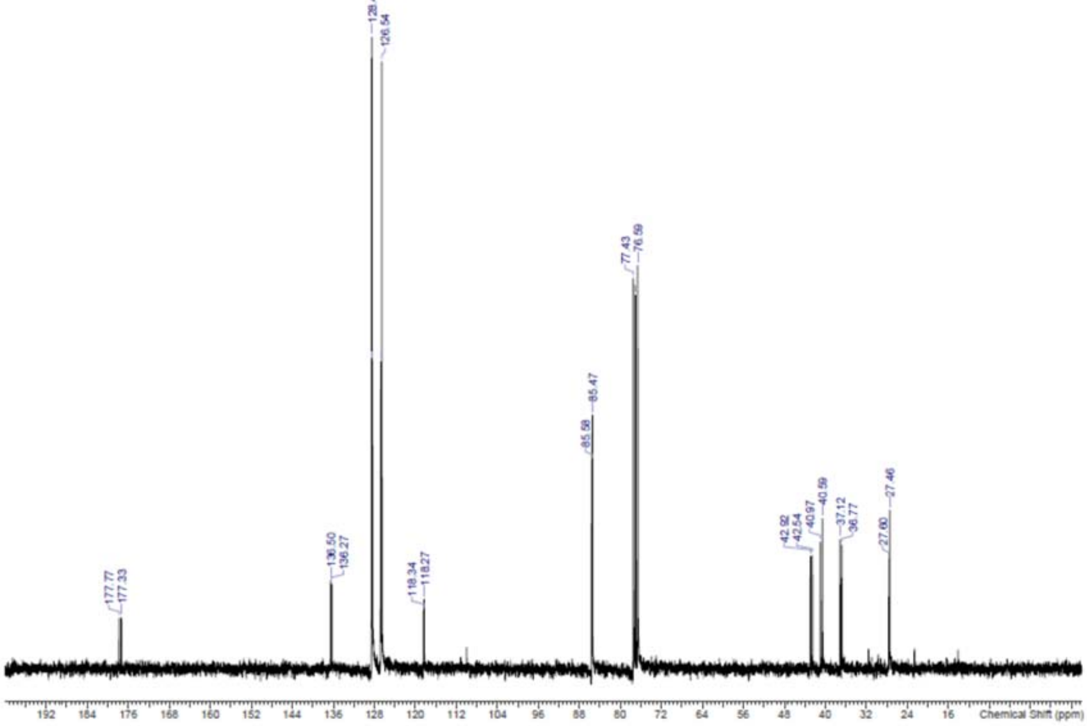

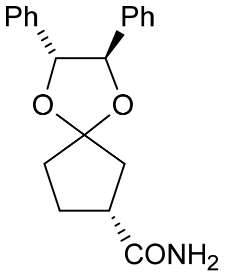

(*R*)-**3a**

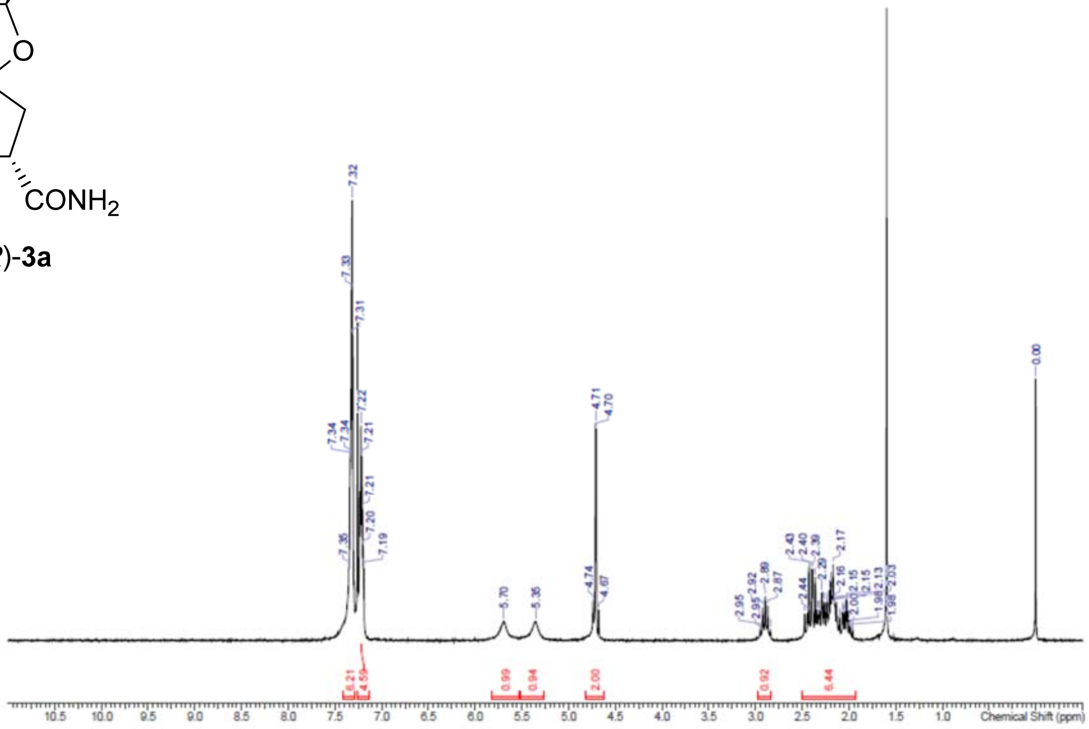

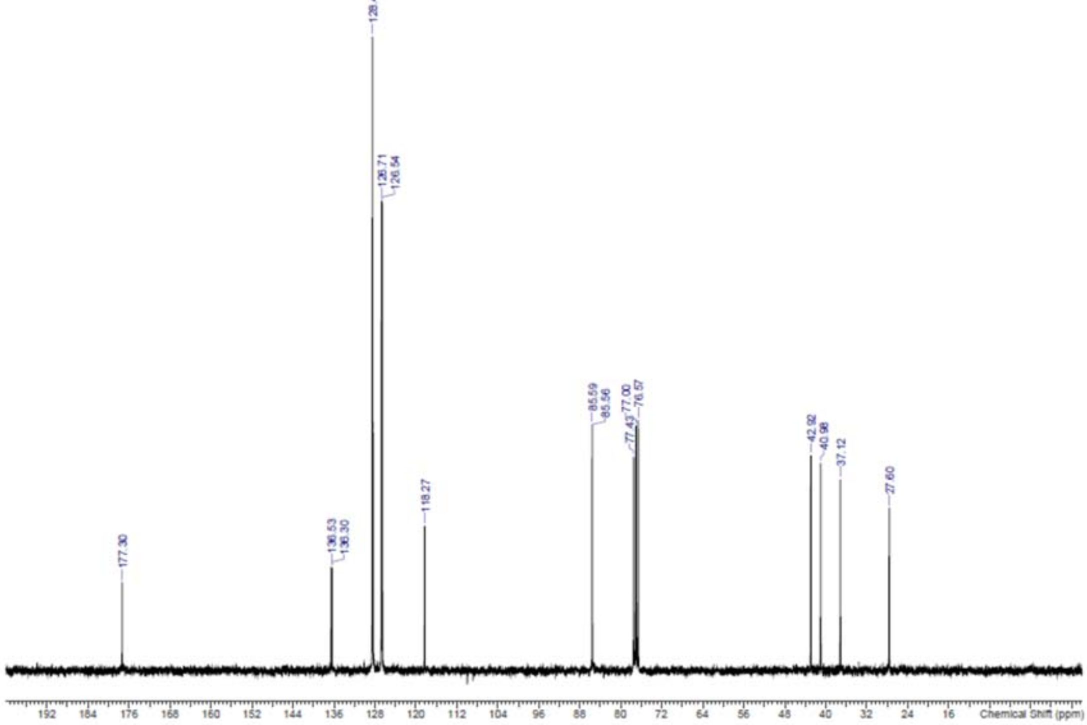

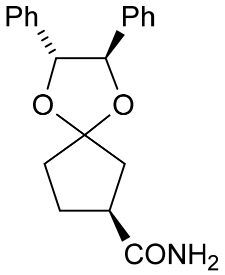
Ph
Ph
O
O
CONH2

(*S*)-**3a**

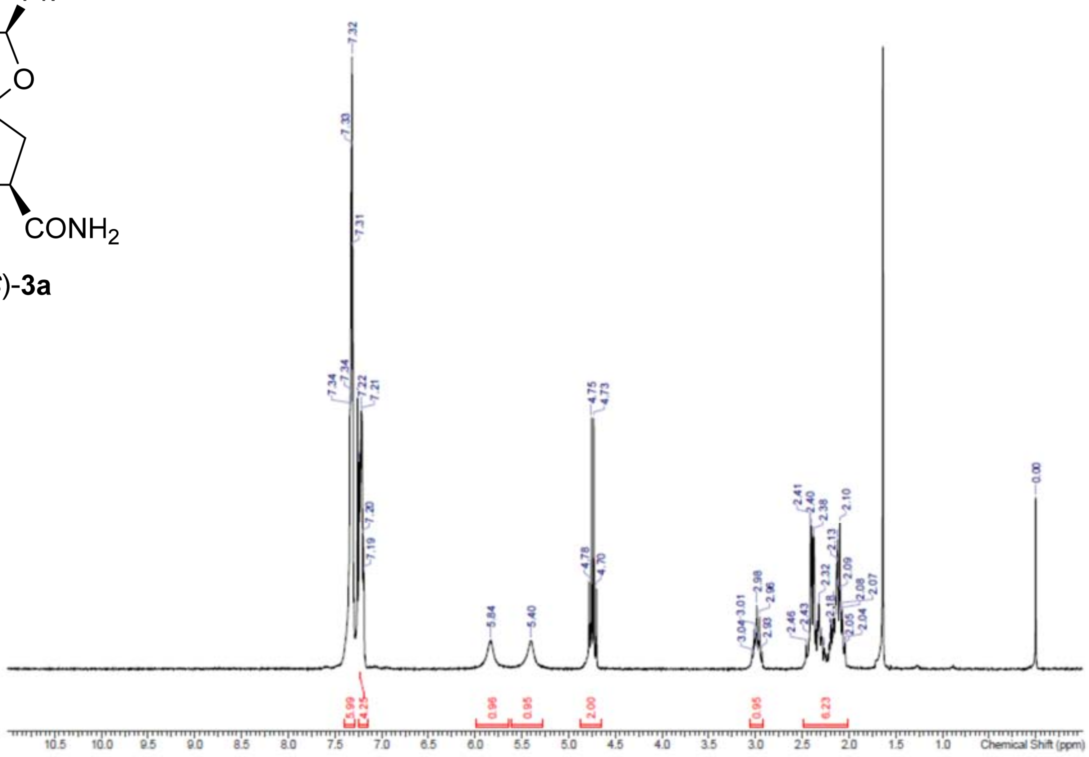
7.34 7.34 7.33 7.32 7.31 7.22 7.21 7.20 7.19
5.84 5.40
4.78 4.75 4.73 4.70
3.04 3.01 2.98 2.96 2.93
2.46 2.43 2.41 2.40 2.38 2.32 2.18 2.13 2.10 2.09 2.08 2.07 2.05 2.04
0.00
5.99 4.25 0.96 0.95 2.00 0.95 6.23
Chemical Shift (ppm)

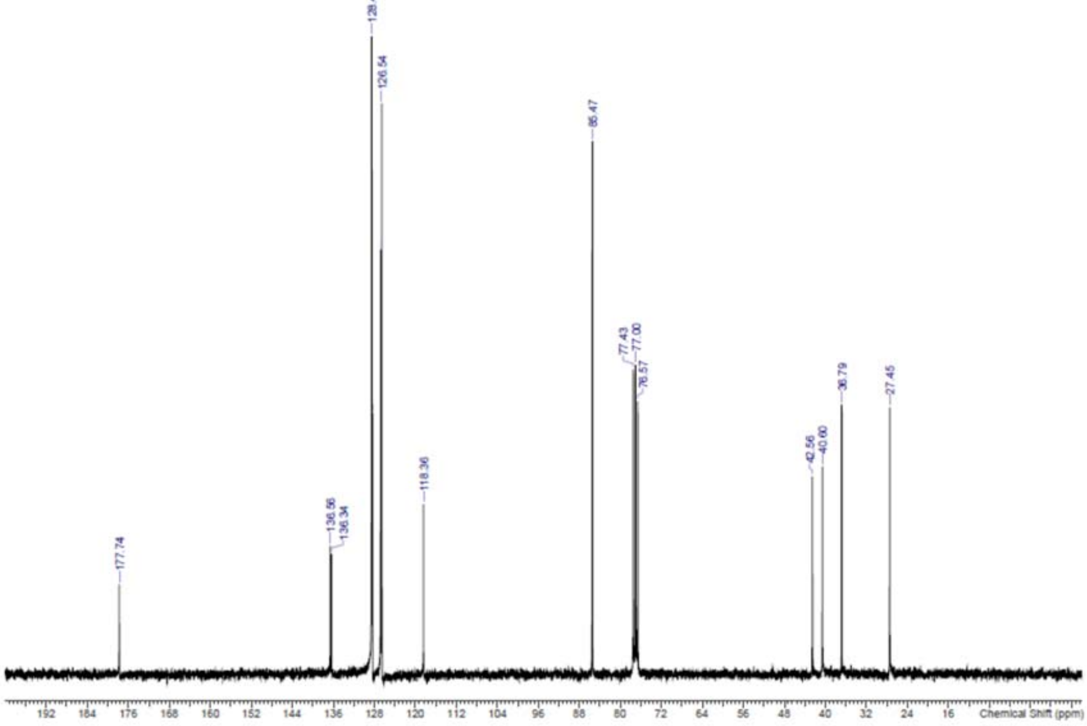
177.74
136.56 136.34
128.49 126.54
118.36
85.47
77.43 77.00 76.57
42.56 40.60 36.79 27.45
Chemical Shift (ppm)

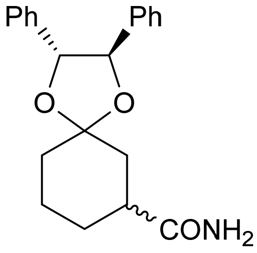
Ph
Ph
O
O
CONH2

3b

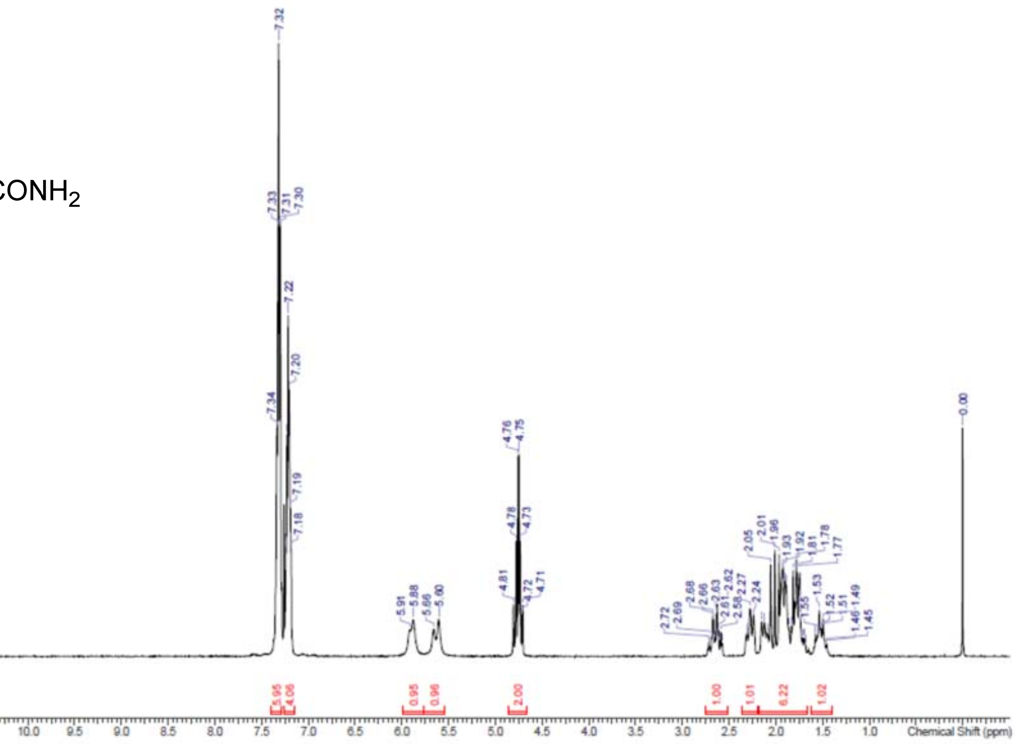
7.32
7.33
7.31
7.30
7.22
7.20
7.34
7.19
7.18
5.91
5.88
5.66
5.60
4.76
4.75
4.78
4.73
4.81
4.72
4.71
2.72
2.69
2.68
2.66
2.63
2.61
2.62
2.58
2.27
2.24
2.05
2.01
1.96
1.93
1.92
1.81
1.78
1.77
1.55
1.53
1.52
1.51
1.46
1.49
1.45
0.00
5.95
4.06
0.95
0.96
2.00
1.00
1.01
6.22
1.02
Chemical Shift (ppm)

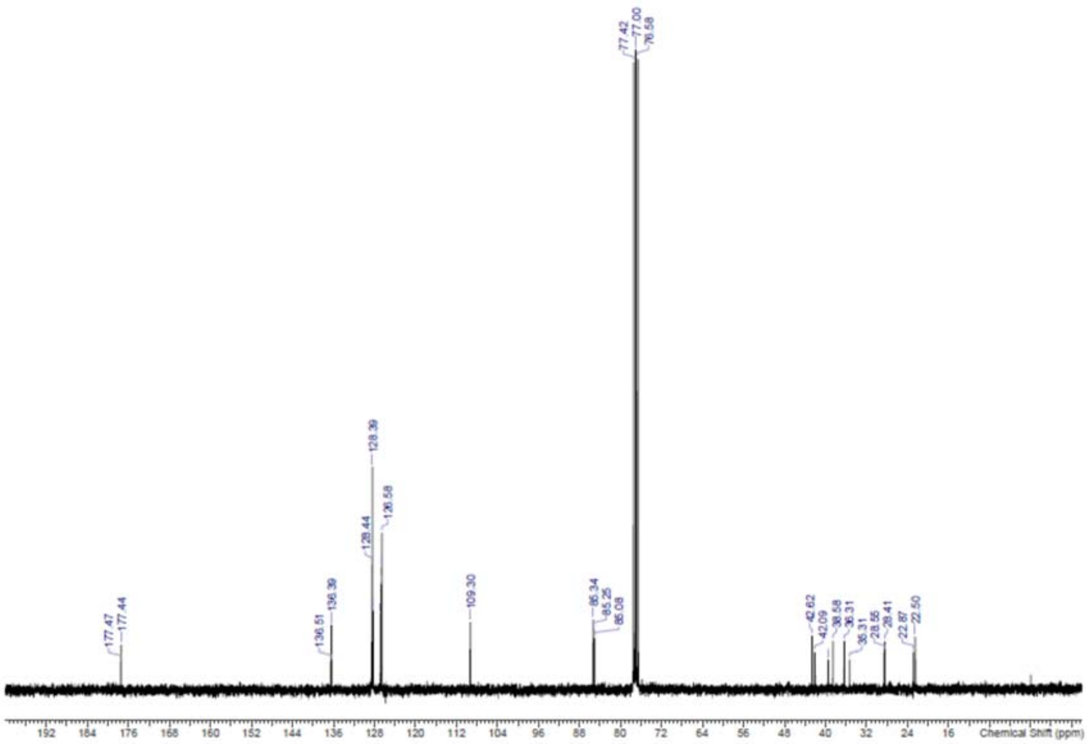
77.42
77.00
76.58
128.39
128.44
126.58
109.30
136.51
136.39
177.47
177.44
85.34
85.25
85.08
42.62
42.09
38.58
36.31
35.31
28.56
28.41
22.87
22.50
Chemical Shift (ppm)

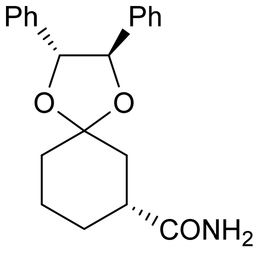
Ph
Ph
O
O
CONH2

(*R*)-**3b**

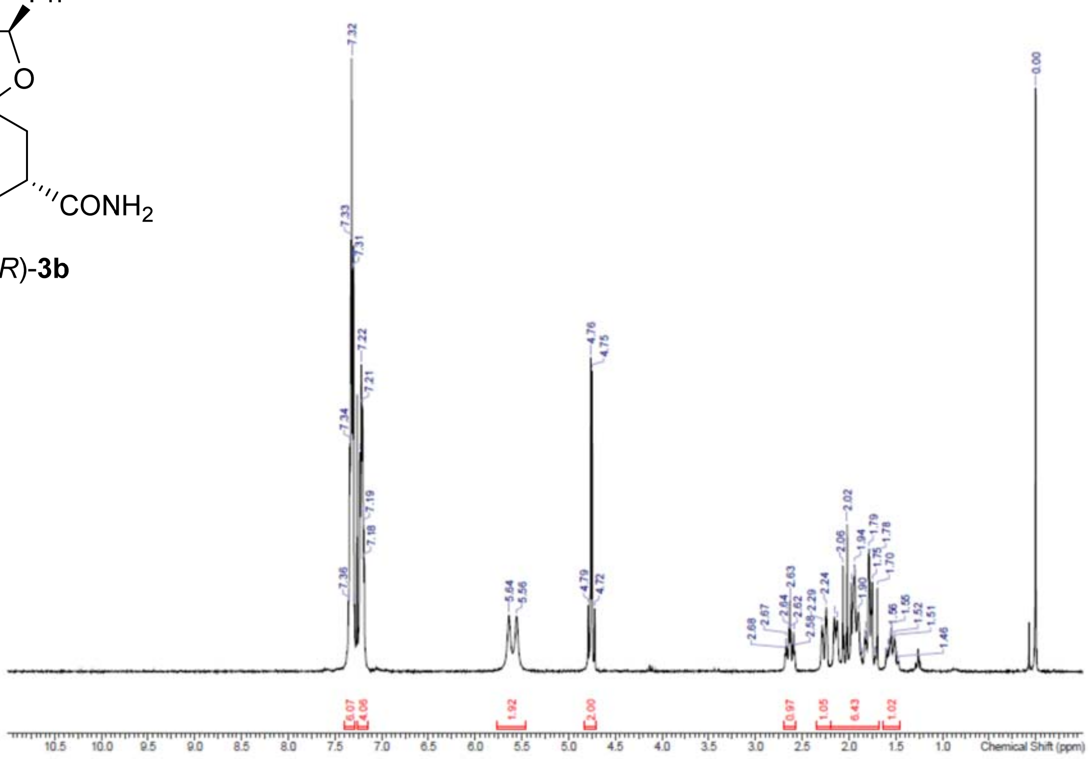
7.32
7.33
7.31
7.22
7.21
7.34
7.19
7.18
7.36
5.64
5.56
4.76
4.75
4.79
4.72
2.68
2.67
2.64
2.63
2.62
2.58
2.29
2.24
2.06
2.02
1.94
1.90
1.79
1.78
1.75
1.70
1.52
1.51
1.46
0.00
6.07
4.06
1.92
2.00
0.97
1.05
6.43
1.02
Chemical Shift (ppm)

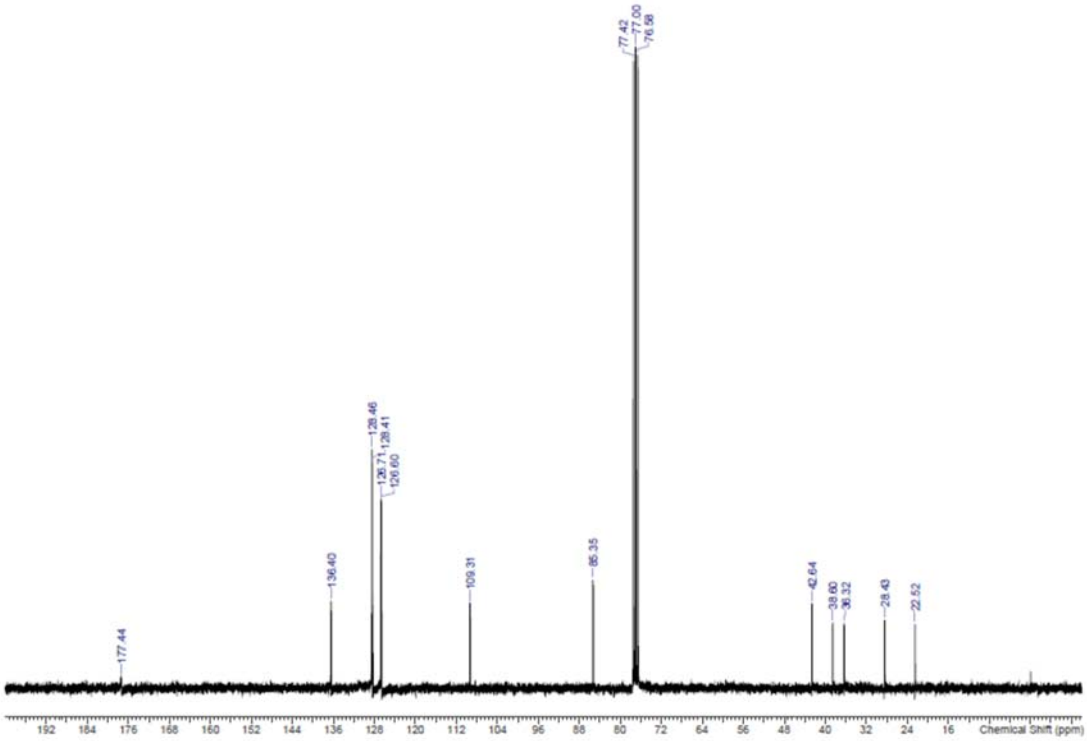
177.44
136.40
128.46
128.41
126.71
126.60
109.31
85.35
77.42
77.00
76.58
42.64
38.60
36.32
28.43
22.52
Chemical Shift (ppm)

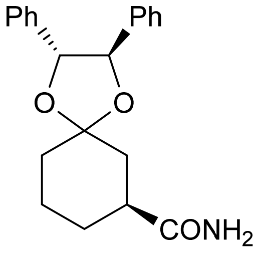
Ph
Ph
O
O
CONH2

(*S*)-**3b**

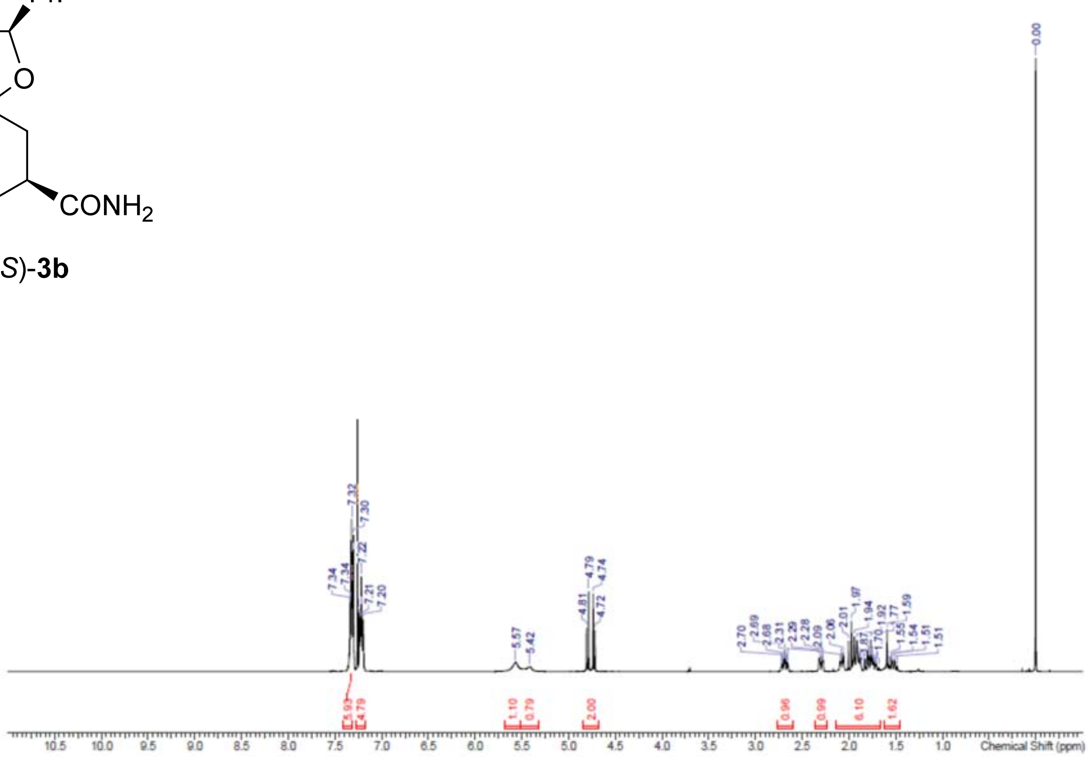
0.00
5.57
5.42
Chemical Shift (ppm)

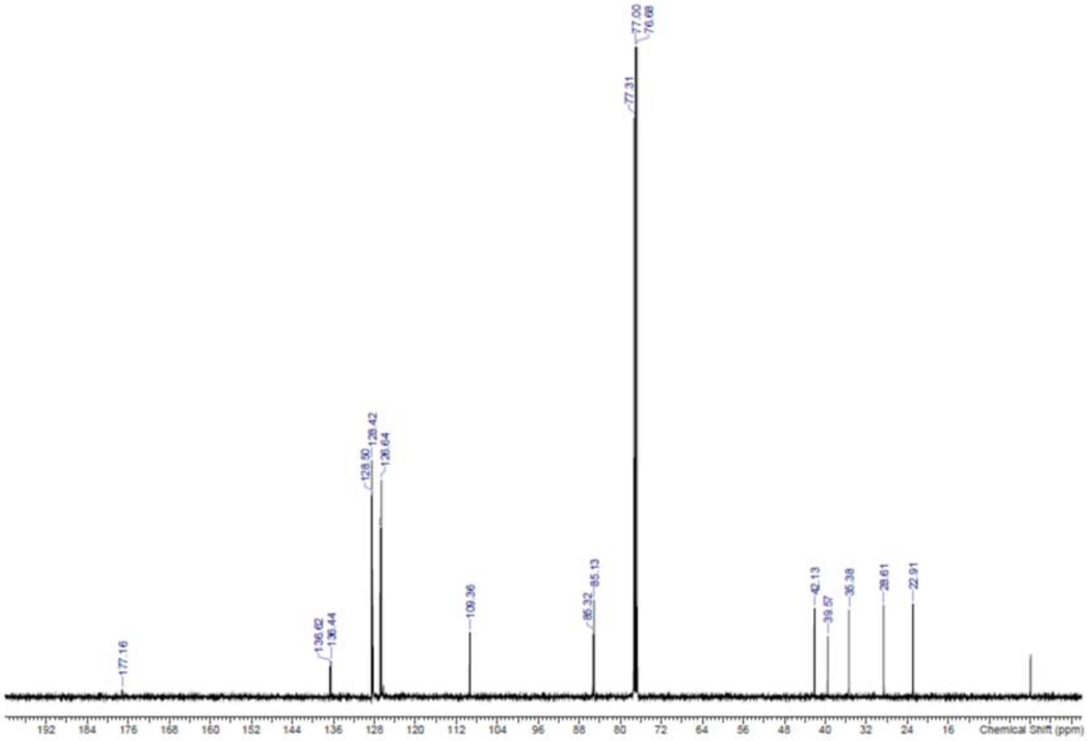
177.16
109.36
85.13
42.13
28.61
22.91
Chemical Shift (ppm)

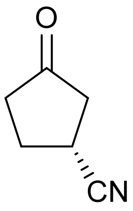
O
CN

(*R*)-**1a**

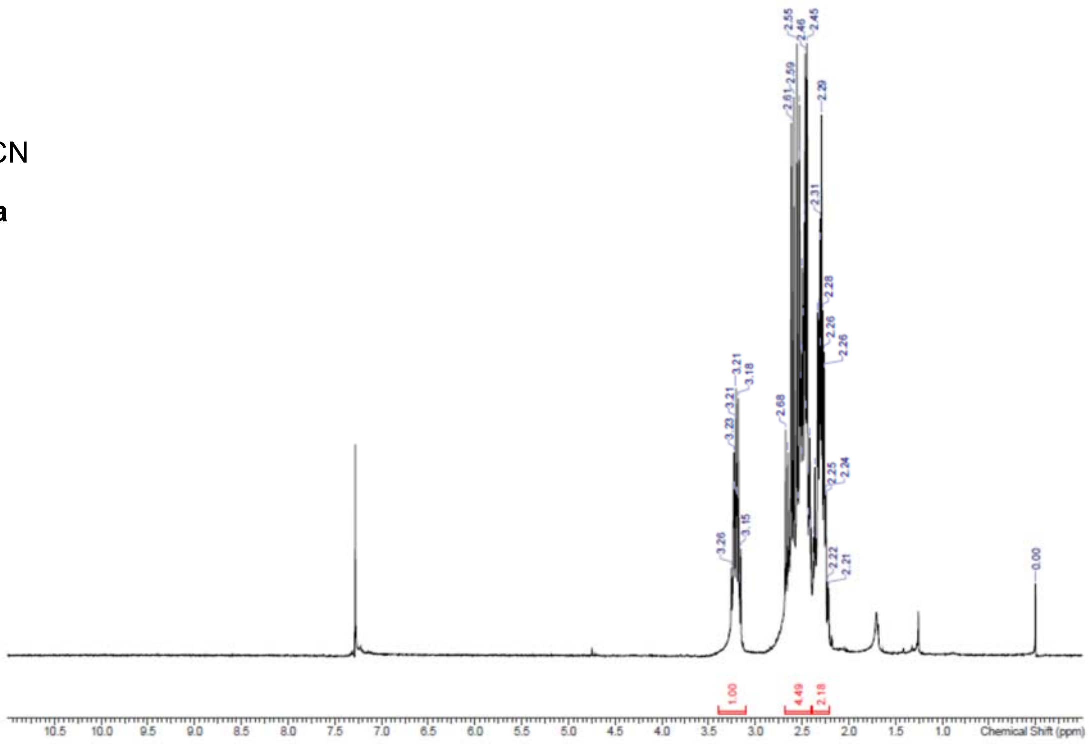
2.55
2.46
2.45
2.61
2.59
2.29
2.31
2.28
2.26
2.26
3.21
3.23
3.21
3.18
2.68
2.25
2.24
3.26
3.15
2.22
2.21
0.00
1.00
4.49
2.18
Chemical Shift (ppm)

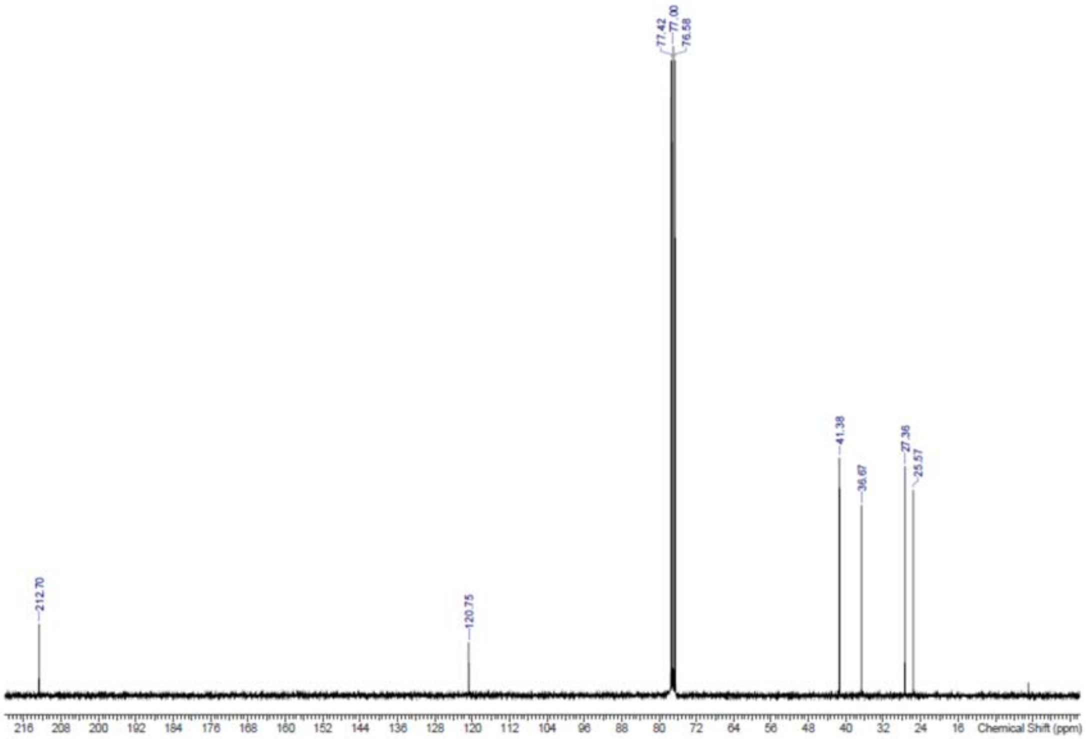
77.42
77.00
76.58
41.38
36.67
27.36
25.57
212.70
120.75
Chemical Shift (ppm)

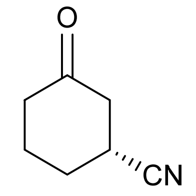
O
CN

(*R*)-**1b**

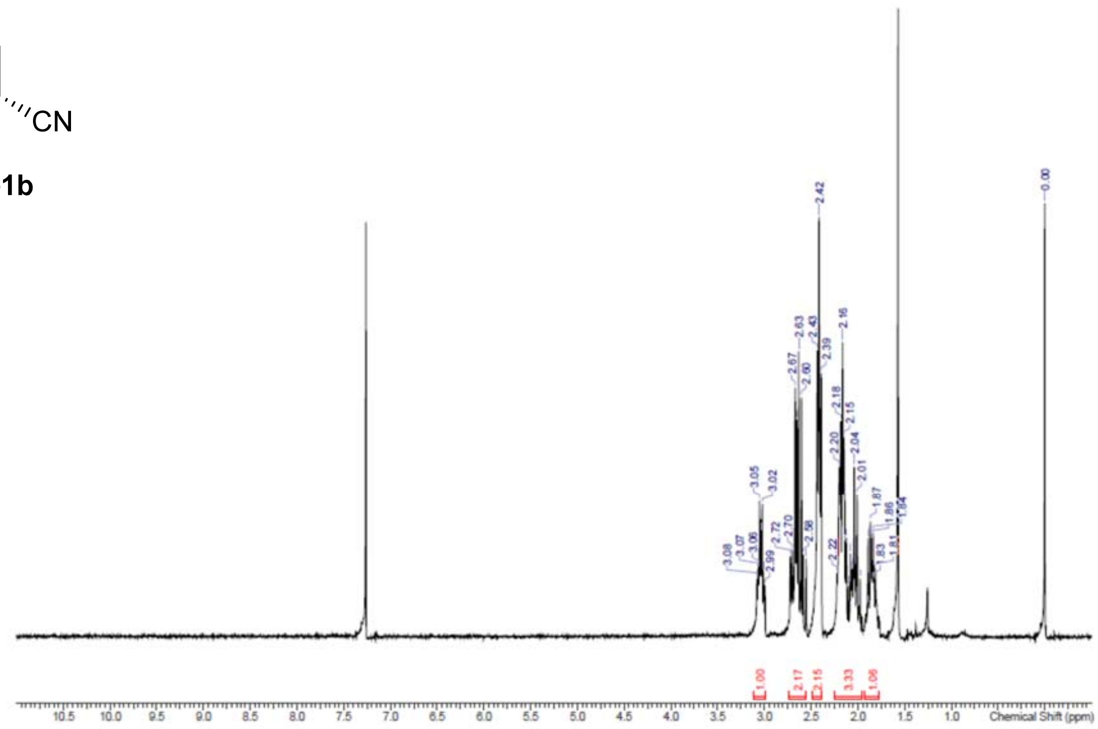
3.08
3.07
3.06
3.05
3.02
2.99
2.72
2.70
2.67
2.63
2.60
2.58
2.43
2.42
2.39
2.22
2.20
2.18
2.16
2.15
2.04
2.01
1.87
1.86
1.84
1.83
1.81
0.00
1.00
2.17
2.15
3.33
1.06
Chemical Shift (ppm)

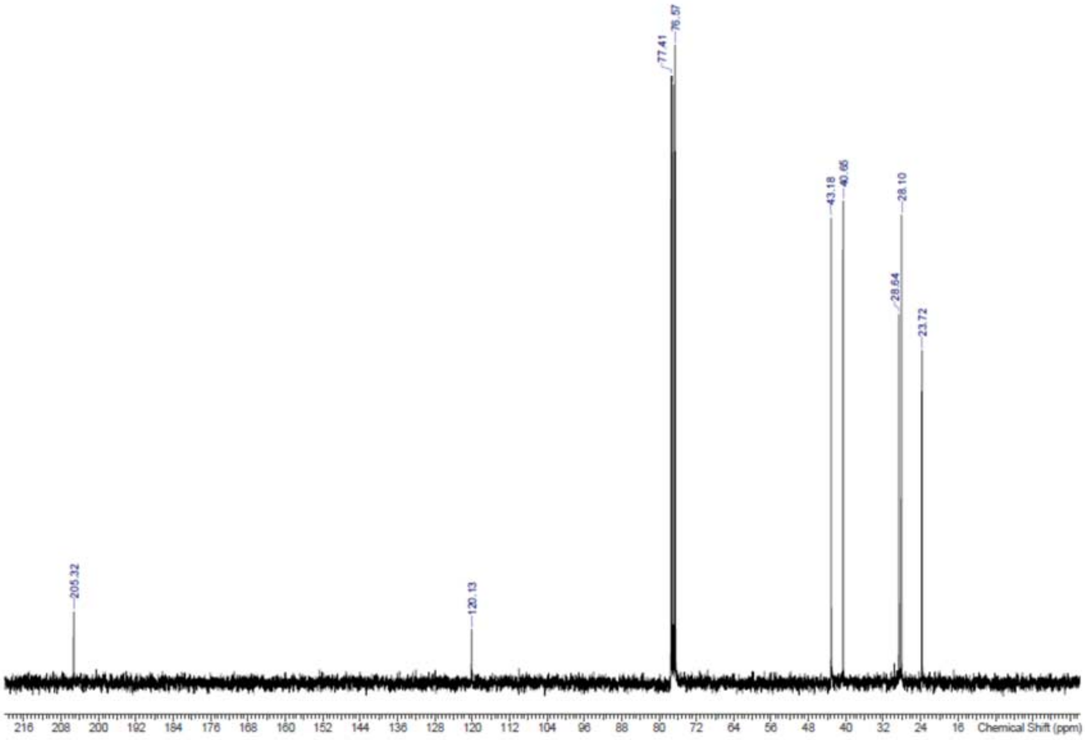
205.32
120.13
77.41
76.57
43.18
40.65
28.64
28.10
23.72
Chemical Shift (ppm)

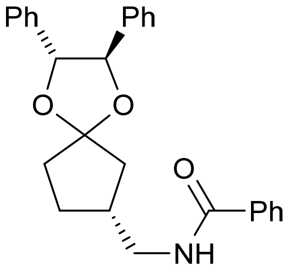

Ph
Ph
O
O
O
Ph
NH

(*R*)-**5a**

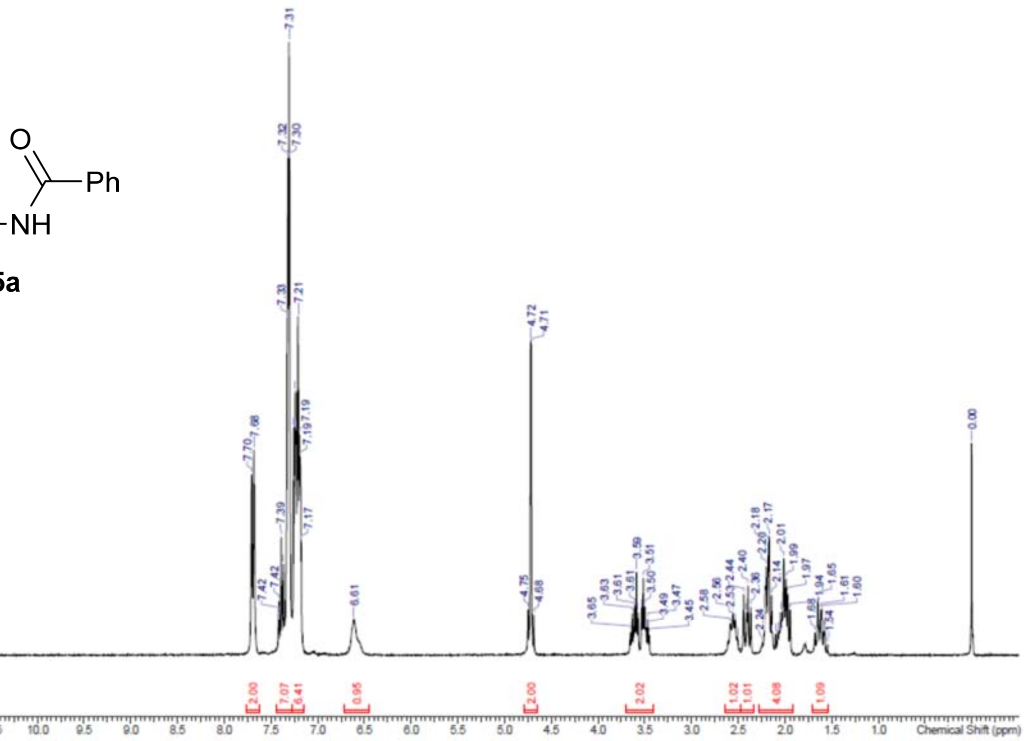

7.31
7.32
7.30
7.33
7.21
7.70
7.68
7.19
7.19
7.39
7.42
7.42
7.17
6.61
4.72
4.71
4.75
4.68
3.59
3.51
3.61
3.63
3.65
3.50
3.49
3.47
3.45
2.18
2.17
2.01
2.20
2.44
2.40
2.56
2.58
2.53
2.36
2.24
2.14
1.99
1.97
1.94
1.65
1.61
1.60
1.68
1.54
0.00
2.00
7.07
6.41
0.95
2.00
2.02
1.02
1.01
4.08
1.09
Chemical Shift (ppm)

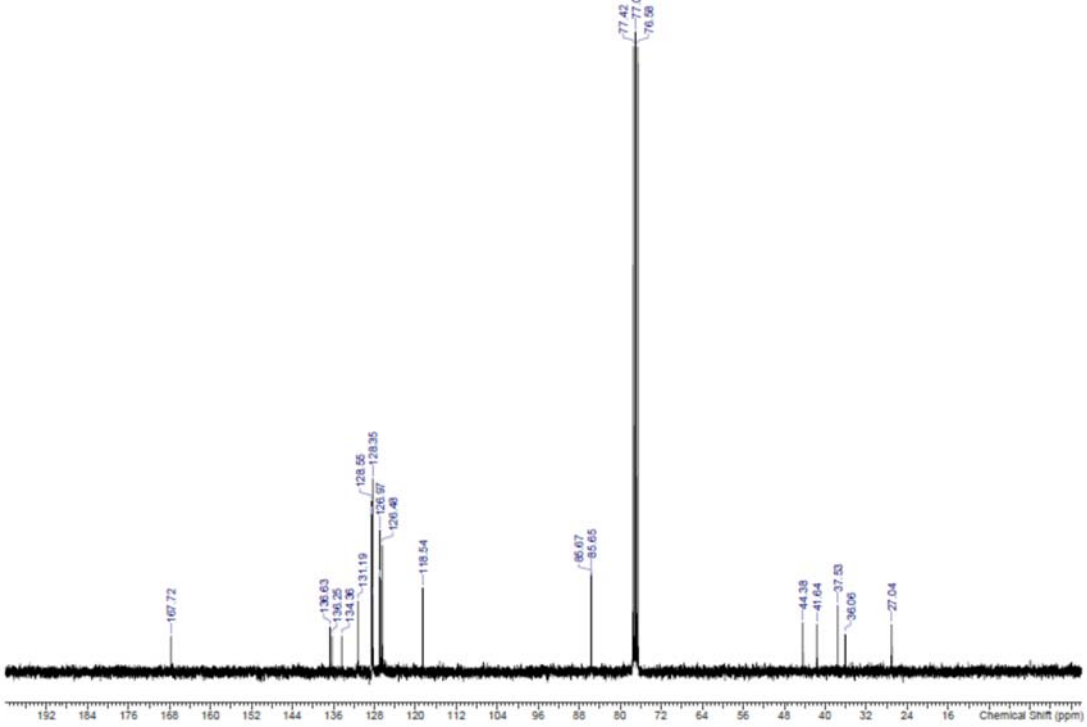

77.42
77.00
76.58
128.55
128.35
126.97
126.48
118.54
131.19
136.63
136.25
134.36
167.72
85.67
85.65
44.38
41.64
37.53
36.06
27.04
Chemical Shift (ppm)

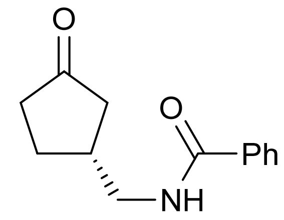
O
O
Ph
NH

(*R*)-**6a**

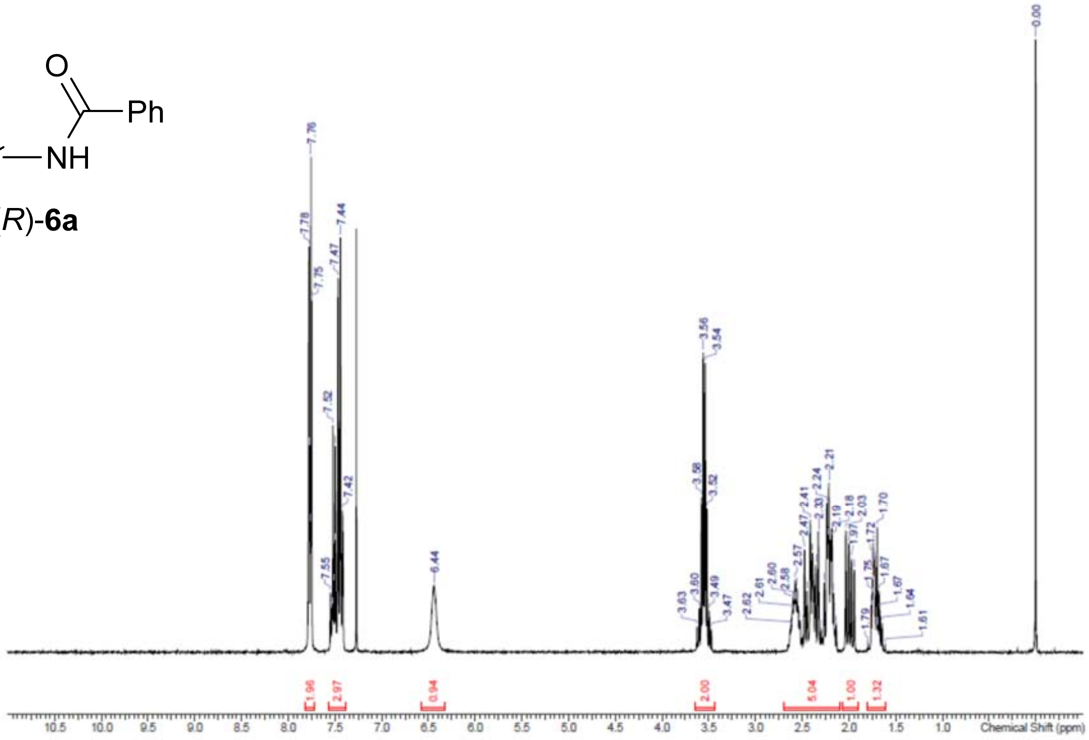
7.78
7.76
7.75
7.55
7.52
7.47
7.44
7.42
6.44
3.63
3.60
3.58
3.56
3.54
3.52
3.49
3.47
2.62
2.61
2.60
2.58
2.57
2.47
2.41
2.30
2.24
2.21
2.19
2.18
2.03
1.97
1.79
1.75
1.72
1.70
1.67
1.64
1.61
0.00
1.96
2.97
0.94
2.00
5.04
1.00
1.32
Chemical Shift (ppm)

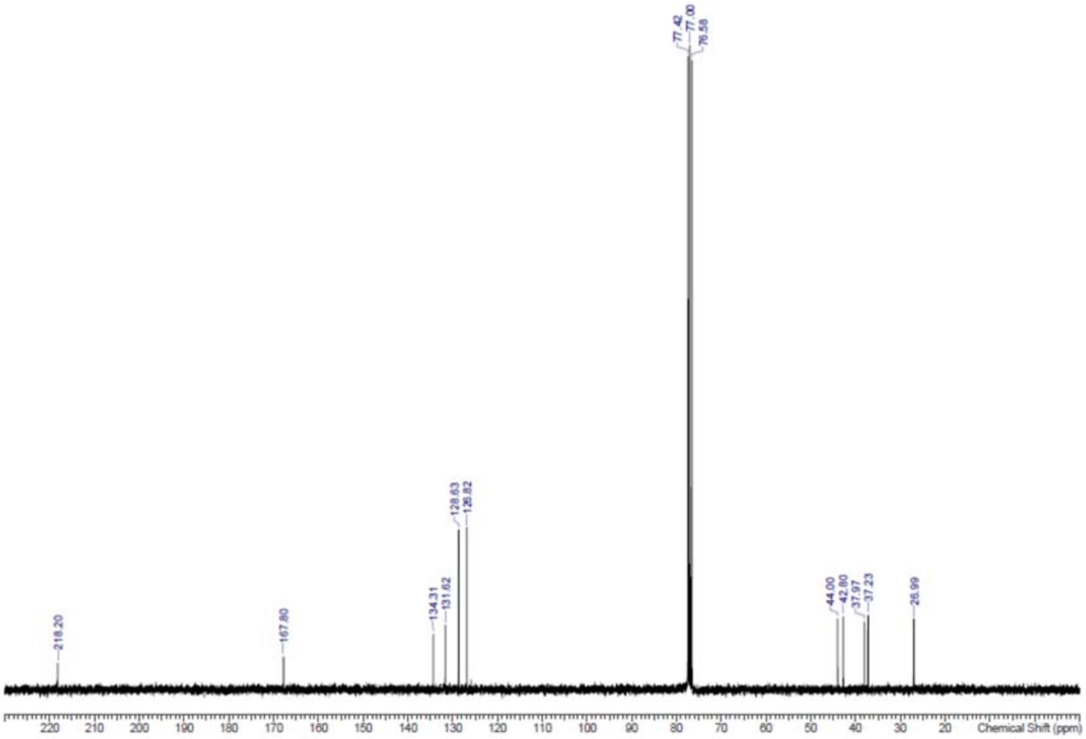
218.20
167.80
134.31
131.62
128.63
126.82
77.42
77.00
76.58
44.00
42.80
37.97
37.23
28.99
Chemical Shift (ppm)

X-Ray crystal structure determination:

Crystals were grown by recrystallization in chloroform-toluene. Single crystals were mounted on a nylon loop with Paratone-N® oil and transferred to the cold gas stream (173 K) of these diffractometers. X-ray crystallographic data of (*R*)-**3a**, (*S*)-**3a**, and (*R*)-**3b** were measured on a Bruker APEXII Ultra CCD diffractometer. Regarding (*S*)-**3b**, the data were collected at a Bruker APEXII CCD diffractometer.

Crystallographic data of (*R*)-**3a** (recrystallized from chloroform-toluene):

$C_{20}H_{21}NO_3$, M = 323.38, monoclinic, space group $P2_1$ (no. 4), a = 6.2977(2), b= 7.4401(2), c = 18.2018(6) Å, β = 92.594(2)°, V = 851.98(5) Å^3, Z = 2, T = 173 K, D_c =1.261 gcm^{-3}, μ(Cu-$K\alpha$) = 0.680 mm^{-1}, F(000)= 344, 9149 measured reflections, 2815 independent, R(int)= 0.0192, 225 parameters were refined against 2815 reflections, R_1 = 0.0304 for 2709 [I > 2σ(I)] and 0.0317 for all data, w$R(F^2)$ = 0.0805 (all data), GOF = 1.073, $\Delta\rho_{min}$ and $\Delta\rho_{max}$= −0.158 and 0.216 e.Å^{-3}. Flack parameter: 0.15(7) The R absolute configuration is assigned by reference to unchanging chiral centers of (1R,2R)-1,2-diphenylethane-1,2-diol used in the synthetic procedure (ketalization). CCDC 1849794 contains crystallographic data.

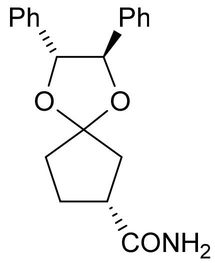

(*R*)-**3a**

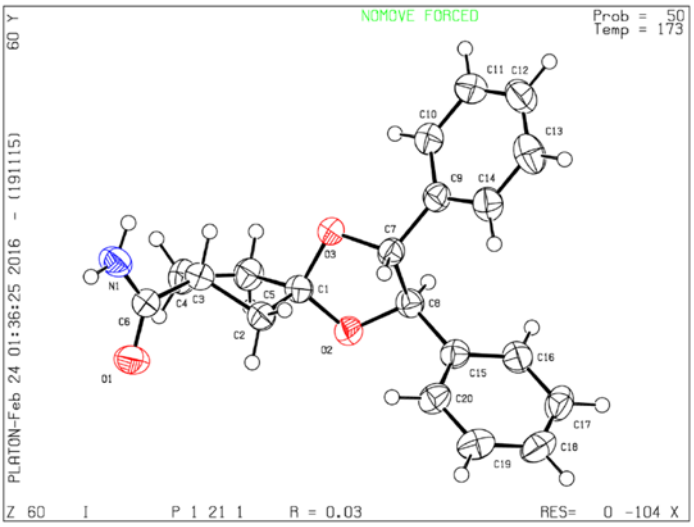

Crystallographic data of (*S*)-**3a** (recrystallized from chloroform-toluene):

$C_{20}H_{21}NO_3$, M = 323.38, monoclinic, space group $P2_1$ (no. 4), a = 6.2884(3), b= 9.3902(5), c = 14.9711(7) Å, β = 98.263(2)°, V = 874.86(7) Å^3, Z = 2, T = 173 K, D_c =1.228 gcm^{-3}, μ(Cu-$K\alpha$) = 0.662 mm^{-1}, F(000)= 344, 8323 measured reflections, 2857 independent, R(int)= 0.0218, 225 parameters were refined against 2857 reflections, R_1 = 0.0359 for 2790 [I > 2σ(I)] and 0.0367 for all data, w$R(F^2)$ = 0.1005 (all data), GOF = 1.035, $\Delta\rho_{min}$ and $\Delta\rho_{max}$= −0.109 and 0.215 e.Å^{-3}. Flack parameter: 0.11(7). The *S* absolute configuration is assigned by reference to unchanging chiral centers of (1*R*,2*R*)-1,2-diphenylethane-1,2-diol used in the synthetic procedure (ketalization). CCDC 1849795 contains crystallographic data.

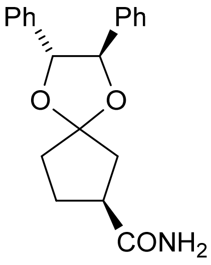

(*S*)-**3a**

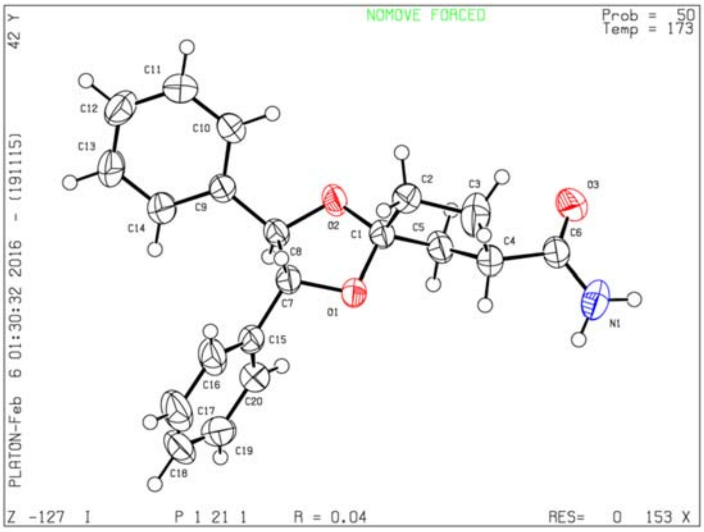

Crystallographic data of (*R*)-**3b** (recrystallized from chloroform-toluene):

$C_{21}H_{23}NO_3$, *M* = 337.40, monoclinic, space group $P2_1$ (no. 4), *a* = 6.4767(4), *b*= 6.7235(5), *c* = 20.8609(14) Å, *β* = 93.851(4)°, *V* = 906.36(11) Å^3, *Z* = 2, *T* = 173 K, D_c =1.236 gcm^{-3}, *μ*(Cu-*K*α) = 0.659 mm^{-1}, F(000)= 360, 6157 measured reflections, 2627 independent, *R*(int)= 0.0241, 226 parameters were refined against 2627 reflections, R_1 = 0.0386 for 2505 [I > 2σ(I)] and 0.0408 for all data, w$R(F^2)$ = 0.0995 (all data), GOF = 1.073, $\Delta\rho_{min}$ and $\Delta\rho_{max}$= −0.133 and 0.193 e.Å^{-3}. Flack parameter: 0.26(12**)**. The *R* absolute configuration is assigned by reference to unchanging chiral centers of (1*R*,2*R*)-1,2-diphenylethane-1,2-diol used in the synthetic procedure (ketalization). CCDC 1849796 contains crystallographic data.

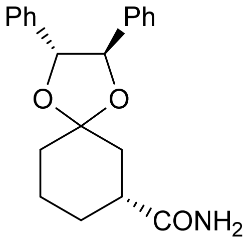

(*R*)-**3b**

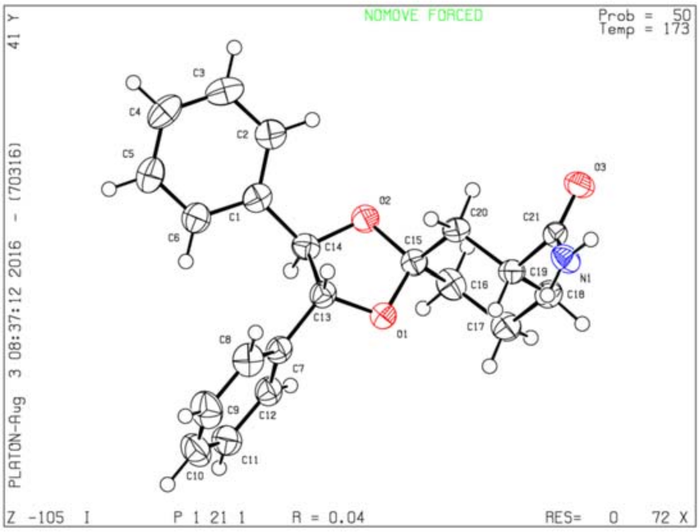

Crystallographic data of (*S*)-**3b** (recrystallized from chloroform-toluene):

$C_{21}H_{23}NO_3$, *M* = 337.40, orthorhombic, space group $P2_12_12_1$ (no. 19), *a* = 9.5492(9), *b*= 9.6708(9), *c* = 19.5238(18) Å, *V* = 1803.0(3) Å^3, *Z* = 4, *T* = 173 K, D_c =1.243 gcm^{-3}, μ(Mo-*K*α) = 0.083 mm^{-1}, F(000)= 720, 10381 measured reflections, 4108 independent, *R*(int)= 0.0244, 226 parameters were refined against 4108 reflections, R_1 = 0.0348 for 3746 [I > 2σ(I)] and 0.0398 for all data, w$R(F^2)$ = 0.0815 (all data), GOF = 1.079, $\Delta\rho_{min}$ and $\Delta\rho_{max}$= −0.133 and 0.193 e.Å^{-3}. Flack parameter: −0.2(4). The *S* absolute configuration is assigned by reference to unchanging chiral centers of (1*R*,2*R*)-1,2-diphenylethane-1,2-diol used in the synthetic procedure (ketalization). CCDC 1849797 contains crystallographic data.

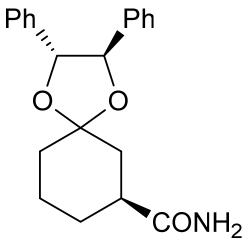

(*S*)-**3b**

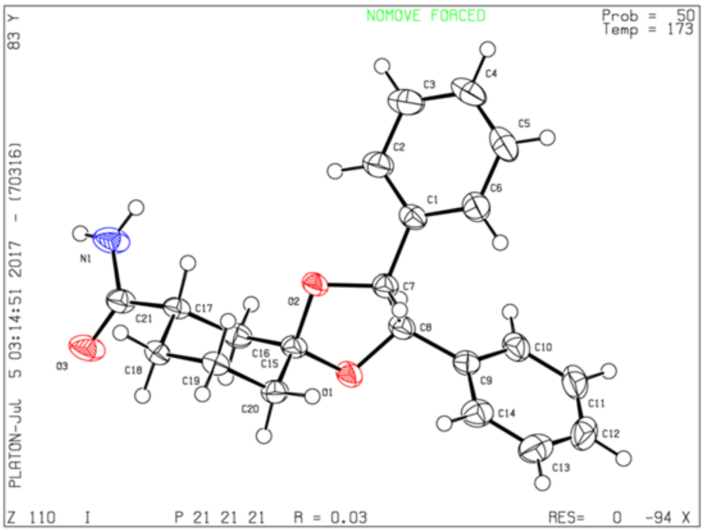

Supplement: RA-008-C8RA06611F-s001 [file RA-008-C8RA06611F-s001.pdf]
